# Calcium binding to a disordered domain of a type III-secreted protein from a coral pathogen promotes secondary structure formation and catalytic activity

**DOI:** 10.1038/s41598-019-42898-0

**Published:** 2019-05-08

**Authors:** Elisabeth Hoyer, Julius Knöppel, Martina Liebmann, Michael Steppert, Manuel Raiwa, Olivia Herczynski, Erik Hanspach, Susanne Zehner, Michael Göttfert, Satoru Tsushima, Karim Fahmy, Jana Oertel

**Affiliations:** 1 Helmholtz-Zentrum Dresden – Rossendorf, Bautzner Landstrasse 400, D-01328 Dresden, Germany; 2 Technische Universität Dresden, Institute of Genetics, Zellescher Weg 20b, D-01217 Dresden, Germany; 3 Leibniz University Hannover, Institute of Radioecology and Radiation Protection, Herrenhäuser Str. 2, D-30419 Hannover, Germany; 4 Present Address: Ludwig-Maximilians-Universität München, Department of Biology I, Microbiology, Großhaderner Str. 2, D-82152 Planegg-Martinsried, Germany

**Keywords:** Molecular biophysics, Biocatalysis, Intrinsically disordered proteins

## Abstract

Strains of the Gram-negative bacterium *Vibrio coralliilyticus* cause the bleaching of corals due to decomposition of symbiotic microalgae. The *V. coralliilyticus* strain ATCC BAA-450 (Vc450) encodes a type III secretion system (T3SS). The gene cluster also encodes a protein (locus tag VIC_001052) with sequence homology to the T3SS-secreted nodulation proteins NopE1 and NopE2 of *Bradyrhizobium japonicum* (USDA110). VIC_001052 has been shown to undergo auto-cleavage in the presence of Ca^2+^ similar to the NopE proteins. We have studied the hitherto unknown secondary structure, Ca^2+^-binding affinity and stoichiometry of the “metal ion-inducible autocleavage” (MIIA) domain of VIC_001052 which does not possess a classical Ca^2+^-binding motif. CD and fluorescence spectroscopy revealed that the MIIA domain is largely intrinsically disordered. Binding of Ca^2+^ and other di- and trivalent cations induced secondary structure and hydrophobic packing after partial neutralization of the highly negatively charged MIIA domain. Mass spectrometry and isothermal titration calorimetry showed two Ca^2+^-binding sites which promote structure formation with a total binding enthalpy of −110 kJ mol^−1^ at a low micromolar K_d_. Putative binding motifs were identified by sequence similarity to EF-hand domains and their structure analyzed by molecular dynamics simulations. The stoichiometric Ca^2+^-dependent induction of structure correlated with catalytic activity and may provide a “host-sensing” mechanism that is shared among pathogens that use a T3SS for efficient secretion of disordered proteins.

## Introduction

The regulation of protein function by Ca^2+^-dependent structural transitions provides a ubiquitous mechanism for cellular signaling networks in all kingdoms of life^1–3^. The rise of intracellular calcium levels is also a typical response in both pathogenic and mutually beneficial plant/microbe interactions^4^. Recently, a potentially virulence-related gene of the prokaryotic coral pathogen *Vibrio coralliilyticus* (VIC_001052, *V. coralliilyticus* strain ATCC BAA-450, Sequence ID: EEX34258.1 Length: 370) has been shown to code for a protein that carries a Ca^2+^-dependent “metal ion-inducible autocleavage” (MIIA) domain^5^. The gene is located in a cluster that encodes a type III secretion system (T3SS). The latter is expressed in a highly controlled manner in a large variety of Gram-negative plant and animal bacterial pathogens^6^. VIC_001052 was discovered by the sequence similarity of its MIIA domain with corresponding domains in NopE1 and NopE2 proteins from *Bradyrhizobium japonicum* USDA110 (Fig. S1), a beneficial symbiont of several legumes. The NopE proteins are T3SS-secreted proteins, which enhance or attenuate symbiosis and exhibit Ca^2+^-dependent auto-cleavage activity^7,8^. The remarkable similarity in sequence and activity between the two domains is contrasted by the biological consequences of microbe activity which lead to symbiosis in plant roots (e.g. soybean) in one case but to “bleaching” of a coral (*Pocillopora damicornis*) in the other^7,9^, which might result from an attack on the algae^10^.

The shared Ca^2+^-dependent autocatalytic activity in the protein domains of the distantly related bacteria raises the question how Ca^2+^ ions regulate the diverse biological functions by presumably similar structural transitions. However, neither the stoichiometry nor the structural basis of Ca^2+^ binding are known. At the molecular level, the most common Ca^2+^-binding protein domain is the EF-hand which exhibits a well-defined helix-turn-helix structure^11^ originally described for parvalbumin^12^. The canonical helix-turn-helix motif typically undergoes tertiary structural changes upon Ca^2+^ binding but maintains its characteristic secondary structure elements also in the metal-free state^13–15^. Related Ca^2+^-binding motifs have been described that are only partially structured^16^ or virtually unstructured^17^ in the absence of Ca^2+^ but fold into an EF-hand upon Ca^2+^ binding. The structural features of the MIIA domain from *V. coralliilyticus* are unexplored and the molecular mechanism of enzyme activity is unknown. With its large content of charged amino acids (42 negatively and 21 positively charged residues in a total of 171 amino acids), the MIIA domain resembles intrinsically disordered proteins (IDPs) as revealed by IDP predictors (Fig. S1) but it does not contain a strict IDP motif^18,19^. Likewise, clusters of carboxyl-carrying amino acids are present that resemble those of Ca^2+^-binding loop in EF-hand motifs but none of these sequences matches precisely the Ca^2+^-binding loop consensus sequence. The lack of classical Ca^2+^-binding motifs in the primary structure of the MIIA domain does not only prevent any prediction of the ion-binding stoichiometry. It also raises the question whether an ion-binding motif is structurally preformed or is induced only in the presence of appropriate metal cations. Here, we have investigated the stoichiometry, affinity and structural consequences of metal ion binding to the MIIA domain using mass spectrometry, calorimetry and CD spectroscopy. Similar to NopE proteins, the MIIA domain of *V*. *coralliilyticus* discriminates between divalent cations, leading to secondary structure formation and catalytic activity for Ca^2+^ and Mn^2+^ but not for Mg^2+^. Due to the lack of more detailed structural data, we further performed homology modelling in combination with molecular dynamics simulations to elucidate whether EF-hand-like Ca^2+^-binding loop structures may partake in Ca^2+^-regulated bacterial pathogenicity or symbiosis.

## Results

### Cation-selective secondary structure formation

**Figure 1:**
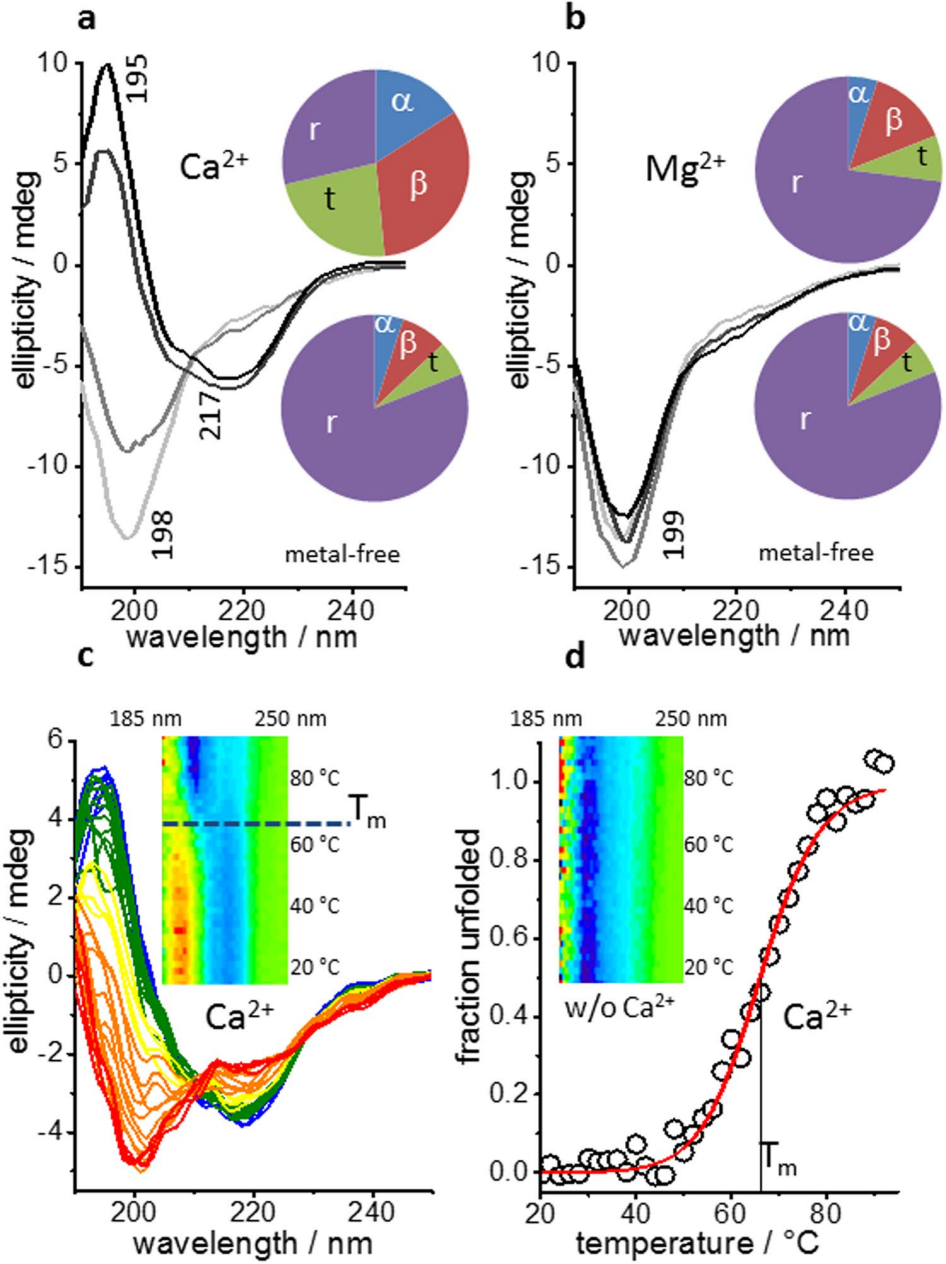
Circular Dichroism spectra of the D116A-MIIA domain as a function of metal cation binding and thermal unfolding. (**a**) CD spectra in the absence of metal ions (light gray) and in the presence of increasing concentrations of Ca^2+^ (100, 250, 500 µM from gray to black). (**b**) Data as in (**a**) for Mg^2+^. **(c)** Series of temperature-dependent CD spectra from 18 to 92 °C in the presence of 100 µM Ca^2+^. Inset: the same data as 2D color plot. (**d**) Fit of the data in (**c**) with a two-state unfolding model using the integrated deviation (in absolute numbers) of the spectra at each temperature from that at 18 °C. The mid temperature of unfolding is T_m_ = 66 °C (horizontal line in **(c)**, inset). Inset: 2D color plot obtained in the absence of Ca^2+^, where the low structure content did not exhibit temperature sensitivity.

The MIIA domain has been shown to undergo metal-dependent autocleavage C-terminal of the aspartic acid residue D116 in the GDPH motif of the wild type (wt-MIIA). This reaction is inhibited by the D116A replacement (D116A-MIIA domain)^5^. We have used the D116A variant to assess the potential of the divalent cations Ca^2+^, Mg^2+^, Mn^2+^ and the trivalent lanthanoids Eu^3+^ and Tb^3+^ to induce secondary structure in the MIIA domain without potential interference from catalytic turnover. Structural transitions were monitored by circular dichroism (CD) spectroscopy. In the absence of metal ions, the D116A-MIIA domain exhibited a negative CD signal at 198 nm, typical of a largely disordered peptide backbone (Fig. 1a). In the presence of 100 µM Ca^2+^, the negative CD band at 198 nm was replaced by a positive band at 195 nm and a negative CD signal appeared at 217 nm, indicative of structure formation. Although we were interested in the relative efficiency of structure induction by different metal cations, rather than in a detailed secondary structure analysis, we show in the pie-charts (Fig. 1, insets) the result of a spectral decomposition generated with Dichroweb (Materials and Methods). The percentage of secondary structure assignments varied with the use of reference datasets and analysis methods but the metal-induced increase in all three secondary structure elements and the concomitant decrease in random coil was consistently observed, irrespective of the evaluation method. In contrast, Mg^2+^ did not cause secondary structure formation (Fig. 1b). In addition to Ca^2+^, also Mn^2+^ and the trivalent lanthanides Eu^3+^ and Tb^3+^ evoked very similar changes in the CD signals (Fig. S2).

The Ca^2+^-induced structure formation implies an increase in secondary structure-specific H-bonds and possibly other intramolecular contacts which are not necessarily restricted to the actual metal ion-coordinating residues. We have used thermal denaturation to determine the free enthalpy that accompanies disruption of intramolecular contacts during unfolding in the metal-bound state of the D116A-MIIA domain. Figure 1(c) shows a series of CD raw data of the Ca^2+^-bound protein between 18 °C and 92 °C. The transition from the folded to the unfolded state is clearly visible in the spectra and has been analyzed by fitting the integrated spectral changes by a two-state equilibrium unfolding model (Materials and Methods). The resulting sigmoidal could be well reproduced with enthalpic and entropic contributions of ΔH = 153 kJ mol^−1^ (±10 kJ) and ΔS = 452 J mol^−1^ (±30 J mol^−1^), respectively, with a transition midpoint temperature T_m_ = 66 °C (Fig. 1d). The unfolding of the Ca^2+^-bound state was reversible as only little aggregation occurred during unfolding and the room temperature CD spectrum after denaturation was similar in shape and size to that of the initial native state (Fig. S2D). In contrast, the CD spectra in the absence of Ca^2+^ corresponded to mostly disordered states already at room temperature and were virtually unaffected by heating (Fig. 1d, inset).

### Ca^2+^ binding affinity and stoichiometry

**Figure 2:**
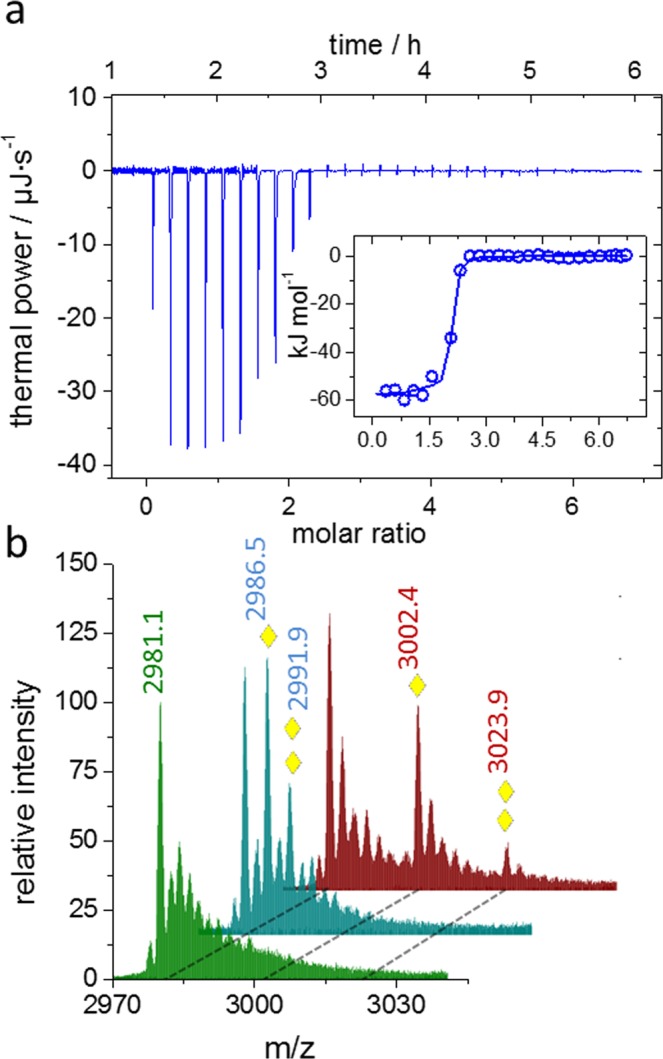
Ca^2+^-binding energy and stoichiometry. (**a**) Isothermal Titration Calorimetry of Ca^2+^ binding to the D116A-MIIA domain. Data were acquired at 25 °C with 54 µM of protein and injection of 10 µL aliquots of 2 mM Ca^2+^ in the presence of 20 mM Tris-SO_4_, pH 7. Two binding sites were fitted with 54 and 57 kJ (±1.1 kJ, and χ^2^ /(degree of freedom) = 1.1). (**b**) Mass spectra representing the +7 charge states of the metal-free D116A-MIIA domain (green) and of the Ca^2+^- (blue) and Eu^3+^-(red) containing samples. Yellow diamonds indicate the shifted main peaks containing one or two bound metal ions.

The spectroscopic data revealed the pronounced structural influence of Ca^2+^ (as well as Mn^2+^, Eu^3+^ and Tb^3+^, Fig. S1) on the MIIA domain at saturating conditions. The dissociation constant and stoichiometry was determined for Ca^2+^, the physiologically most relevant metal, using isothermal titration calorimetry (ITC). As shown in Fig. 2(a), Ca^2+^-binding to the D116A-MIIA domain occurred exothermally with a molar ratio of two Ca^2+^-ions per protein. The data were modeled (VPITC-Origin Software) with two binding sites, exhibiting binding enthalpies between −57 and −54 kJ mol^−1^ and binding entropies between −38 and −59 J mol^−1^ K^−1^, corresponding to K_d_ ≤ 3.6 µM (±1.2 µM). The negative binding entropy agreed well with the demonstrated increase in secondary structure upon Ca^2+^.

The revealed metal-binding stoichiometry is not only important in the physiological context but also crucial for narrowing down the hitherto unknown sequence motifs that may confer metal sensitivity to the MIIA domain in the first place. Therefore, the metal-binding stoichiometry was additionally studied by mass spectrometry. For this purpose, the D116A-MIIA domain was transferred into a volatile ammonium acetate buffer in the presence of either 20 µM Ca^2+^ or Eu^3+^. Figure 2(b) compares the mass spectra of metal-free protein with Ca^2+^- and Eu^3+^-bound states. All three samples show the same base peak at m/z = 2981,1 representing the metal-free D116A-MIIA domain at the predominant +7 charge state. In the presence of both, Ca^2+^ and Eu^3+^, two additional peaks appear in the spectra found at m/z = 2986,5 and m/z = 2991,9 for the Ca^2+^ containing solution and at m/z = 3002,4 and m/z = 3023,9 for the Eu^3+^ containing solution, respectively. The observed mass shifts agree with the expected shift caused by one or two of the respective metal cations. The mass spectra clearly demonstrate the presence of two prominent metal-binding sites for Ca^2+^ in line with the ITC experiment. Moreover, the preservation of the stoichiometry upon substitution of Eu^3+^ for Ca^2+^ supports the formation of a structurally well-defined cation-binding site which, similar to other Ca^2+^-binding proteins, also accepts Eu^3+^ as a surrogate for Ca^2+^ ^20–22^. Given the consistency of the effects of Ca^2+^ and Eu^3+^ on the mass spectra and on the CD curves (Figs 1 and S2), the two ions very likely occupy the same two binding sites. The two studied lanthanides also evoked similar structural transitions in the protein but induced the formation of α-helices less efficiently than Ca^2+^, thereby, leaving a larger fraction of the protein disordered (Fig. S2b,c).

### Metal ion-induced folding and catalytic activity

**Figure 3:**
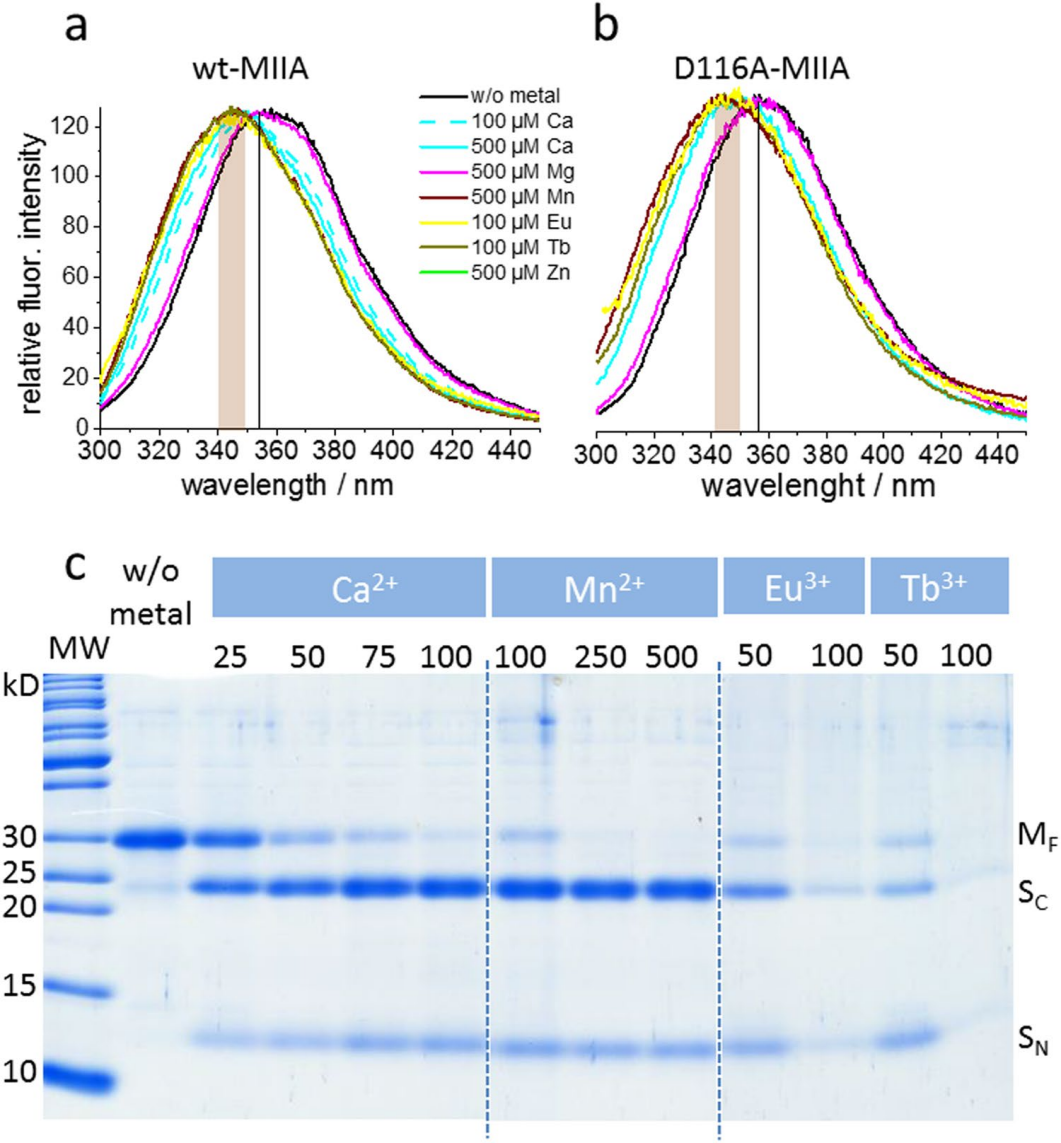
Metal ion-dependent hydrophobic clustering and catalytic activity. Emission spectra of the wild-type (**a**) and D116A-MIIA domain (**b**) in the absence and presence of the indicated cations. The vertical line and shaded area designate the peak wavelength of the metal-free state and the wavelength range of the emission maxima of the states with saturating cation concentrations,respectively. (**c**) Coomassie-blue-stained SDS-PAGE gel showing the appearance of C- (S_C_) and N-terminal (S_N_) protein fragments as a result of metal-ion-induced autocleavage of the MIIA domain (M_F_). The cations and their concentrations (µM) for catalytic activation are plotted above each lane of the gels. All lanes are from the entire gel shown in Fig. S4 (**a**). Only the most relevant sections (separated by broken lines) are displayed here.

We have asked whether the spectroscopically observed Ca^2+^-induced structure formation complies with a folding mechanism that is accompanied by burial of hydrophobic residues, a mechanism typically found for folding of water-soluble proteins and referred to as “hydrophobic collapse”. The MIIA domain carries two native tryptophan residues (W172 and W223) which could be used as intrinsic fluorescent monitors of metal-ion-induced folding. The fluorescence emission maximum of both the wild type and the D116A-MIIA domain shifted from 357 to 349 nm when Ca^2+^ was added (Fig. 3) in agreement with the transfer of one or both tryptophans from a hydrophilic to a more hydrophobic environment upon ion-induced folding. Mn^2+^ and the lanthanides Tb^3+^ and Eu^3+^ caused similar blue shifts of the tryptophan emission, whereas Mg^2+^ showed no effect. For the wild-type protein, the catalytic activity was addressed by sodium dodecyl sulfate polyacrylamide gel electrophoresis (SDS-PAGE) after the spectroscopic experiments to reveal the appearance of the N- and C-terminal cleavage fragments S_N_ and S_C_, respectively. In agreement with efficient cleavage at 100 µM Ca^2+^, also the 100 µM Ca^2+^-induced emission shift was of the same size as that evoked by 500 µM (Fig. 3a). At 50 µM Eu^3+^ or Tb^3+^, an emission shift comparable to that in the presence of 100 µM Ca^2+^ and catalytic activity was observed with both cations as well. This supports the native-like interaction of the lanthanides with the Ca^2+^-binding sites despite less efficient helical structure formation.

**Figure 4:**
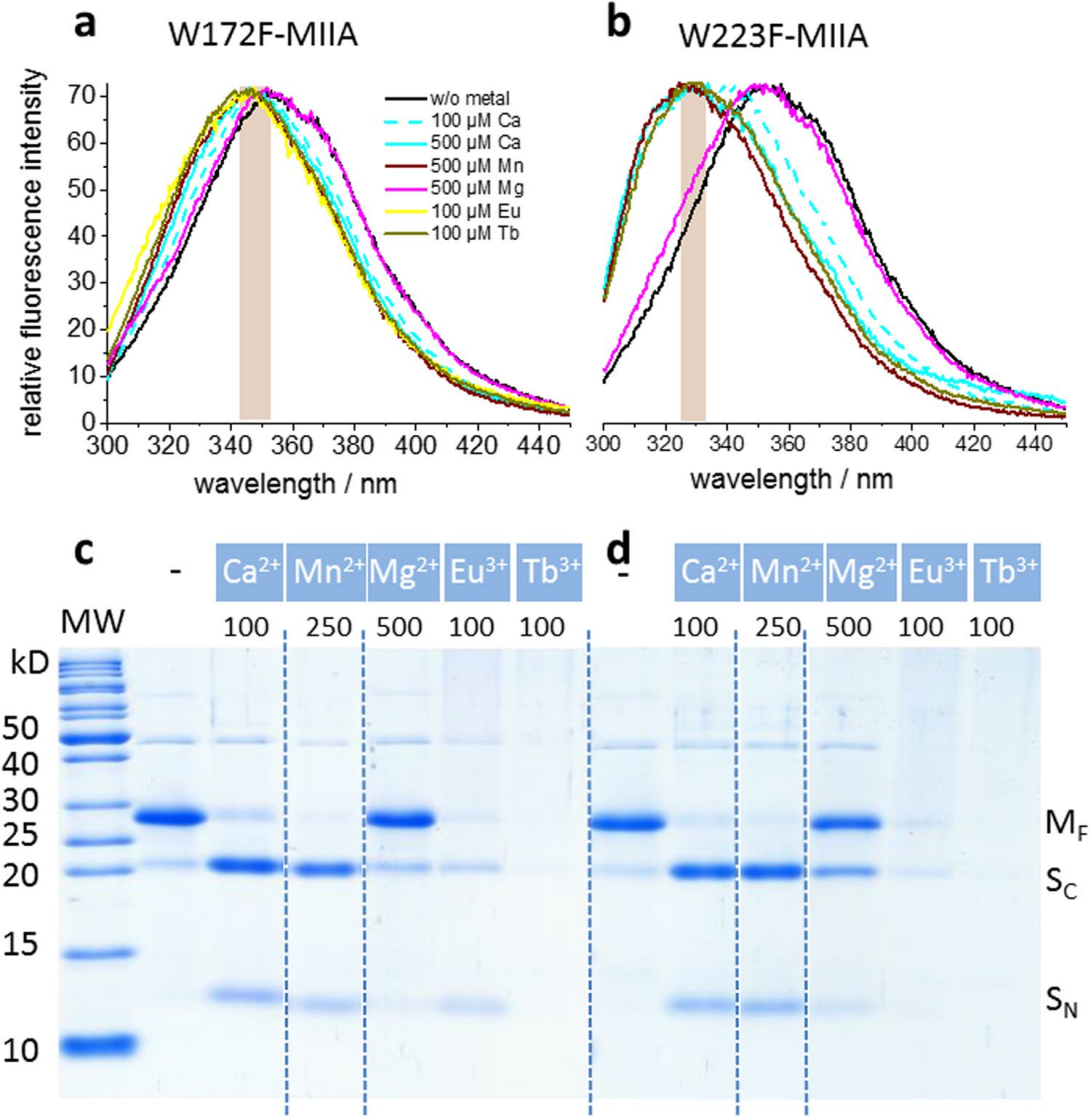
Metal ion-dependent hydrophobic clustering and catalytic activity in single tryptophan replacement variants of the MIIA domain. Emission spectra of the W172F-MIIA (**a**) and W223F-MIIA domain (**b**) in the absence and presence of the indicated cations. The vertical line and shaded area designate the peak wavelength of the metal-free state and the wavelength range of the emission maxima of the states with saturating cation concentrations, respectively. (**c**) and (**d**) Coomassie-blue-stained SDS-PAGE gel showing the appearance of S_C_ and S_N_ protein fragments as a result of metal-ion-induced autocleavage of the W172F-MIIA and W223F-MIIA domain (M_F_), respectively. The cations and their concentrations (µM) for catalytic activation are plotted above each lane of the gels. All lanes in (**c**) and (**d**) are from the entire gels shown in Fig. S5 (**a**) and (**b**), respectively. Only the most relevant sections (separated by broken lines) are displayed here.

The individual fluorescence contributions of the intrinsic tryptophans were further studied by replacing W172 and W223 individually by phenylalanine in the background of the catalytically active wt-MIIA domain (W172F- and W223F-MIIA domain, respectively). In both cases, the metal ions induced hydrophobic clustering of the remaining single tryptophan (Fig. 4a,b) and exhibited secondary structure formation (Fig. S3a,b). Both variants also maintained autocatalytic activity in the presence of Ca^2+^ and Mn^2+^ and trivalent lanthanides (Fig. 4c,d). The replacement of W223 reduced the affinity for Ca^2+^ (compare the emission shifts induced by 100 µM versus 500 µM ion concentration in the two mutants (Fig. 4a,b) but supported more efficient hydrophobic packing of W172 (emission peak at shorter wavelength) than in the wt-MIIA (Fig. 3a).

**Figure 5:**
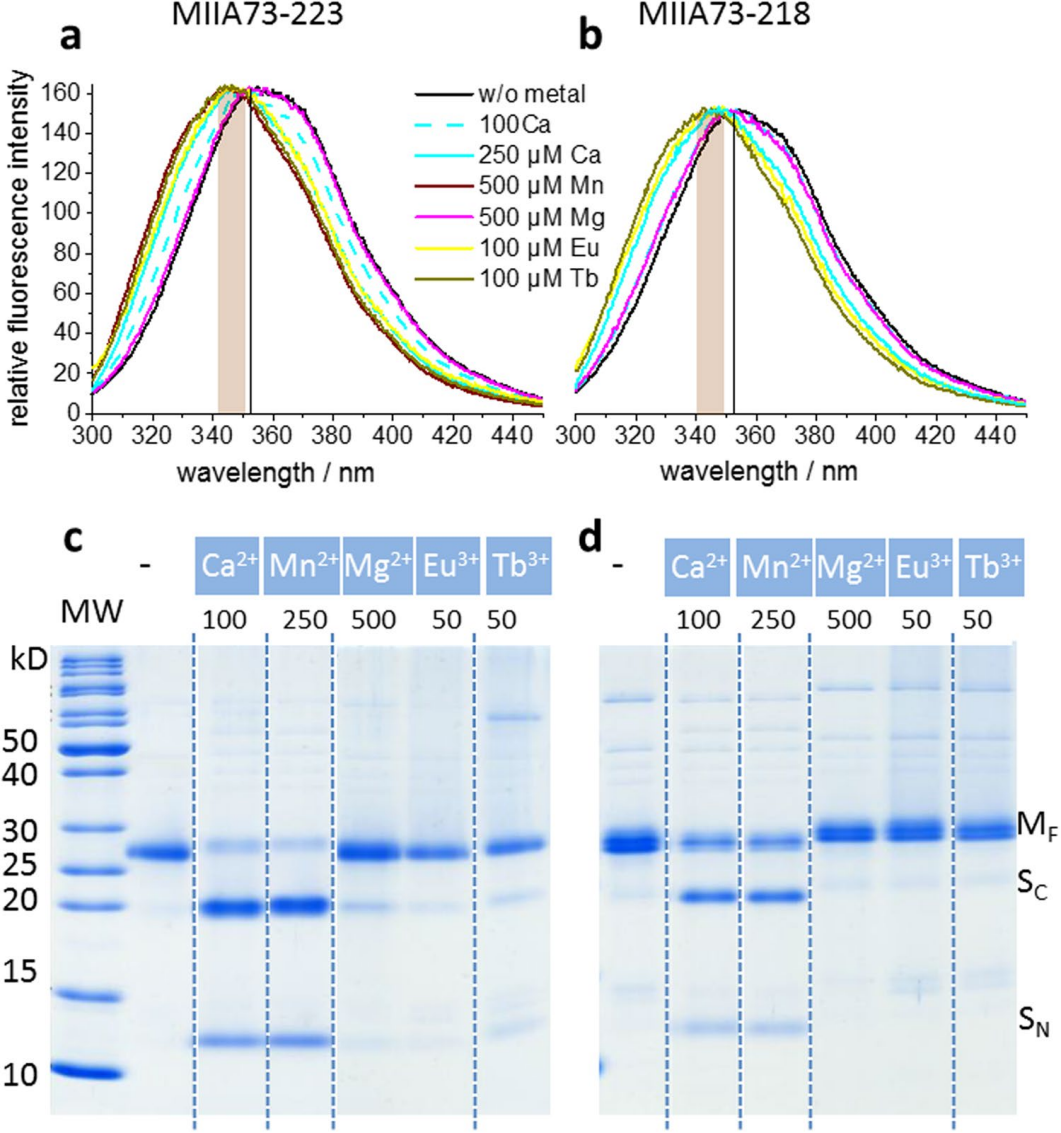
Metal ion-dependent hydrophobic clustering and catalytic activity in C-terminal truncated MIIA derivatives. Emission spectra of the MIIA73-223 (**a**) and MIIA73-218 derivatives (**b**) in the absence and presence of the indicated cations. The vertical line and shaded area designate the peak frequencies of the metal-free and cation-bound states, respectively. (**c**) and (**d**) Coomassie-blue-stained SDS-PAGE gels showing the intact MIIA domains (M_F_) and the appearance of S_C_ and S_N_ protein fragments as a result of metal ion-induced autocleavage of MIIA73-223 **c**) and MIIA73-218 (**d**). The cations and their concentrations (µM) for catalytic activation are plotted above each lane of the gels. All lanes in (**c**) and (**d**) are from the entire gels shown in Fig. S6 (**a**) and(**b**), respectively. Only the most relevant sections (separated by broken lines) are displayed here.

The strong metal-induced shielding of W172 from the aqueous environment suggested particularly efficient hydrophobic packing in the central sequence of the MIIA-domain. This raised the question whether the more distant C-terminal W223 is needed for folding at all. We have expressed C-terminally truncated mutants covering amino acid 73 to 223 (MIIA73-223) and 73 to 218 (MIIA73-218). Both truncated derivatives folded with the above studied metal ions as seen by their CD spectra (Fig. S3c,d) and both exhibited hydrophobic packing and at least partial catalytic activity (Fig. 5). However, Ca^2+^ induced hydrophobic packing less efficiently since 100 µM did not achieve full tryptophan emission shift in MIIA73-223 (Fig. 5a) and was entirely ineffective in MIIA73-218 (Fig. 5b). The two lanthanides barely caused catalytic activity in either truncation mutant at 50 µM (Fig. 5c,d) whereas autocleavage occurred in the wild-type protein.

### Assessment of local structure stabilization upon Ca^2+^-binding

**Figure 6:**
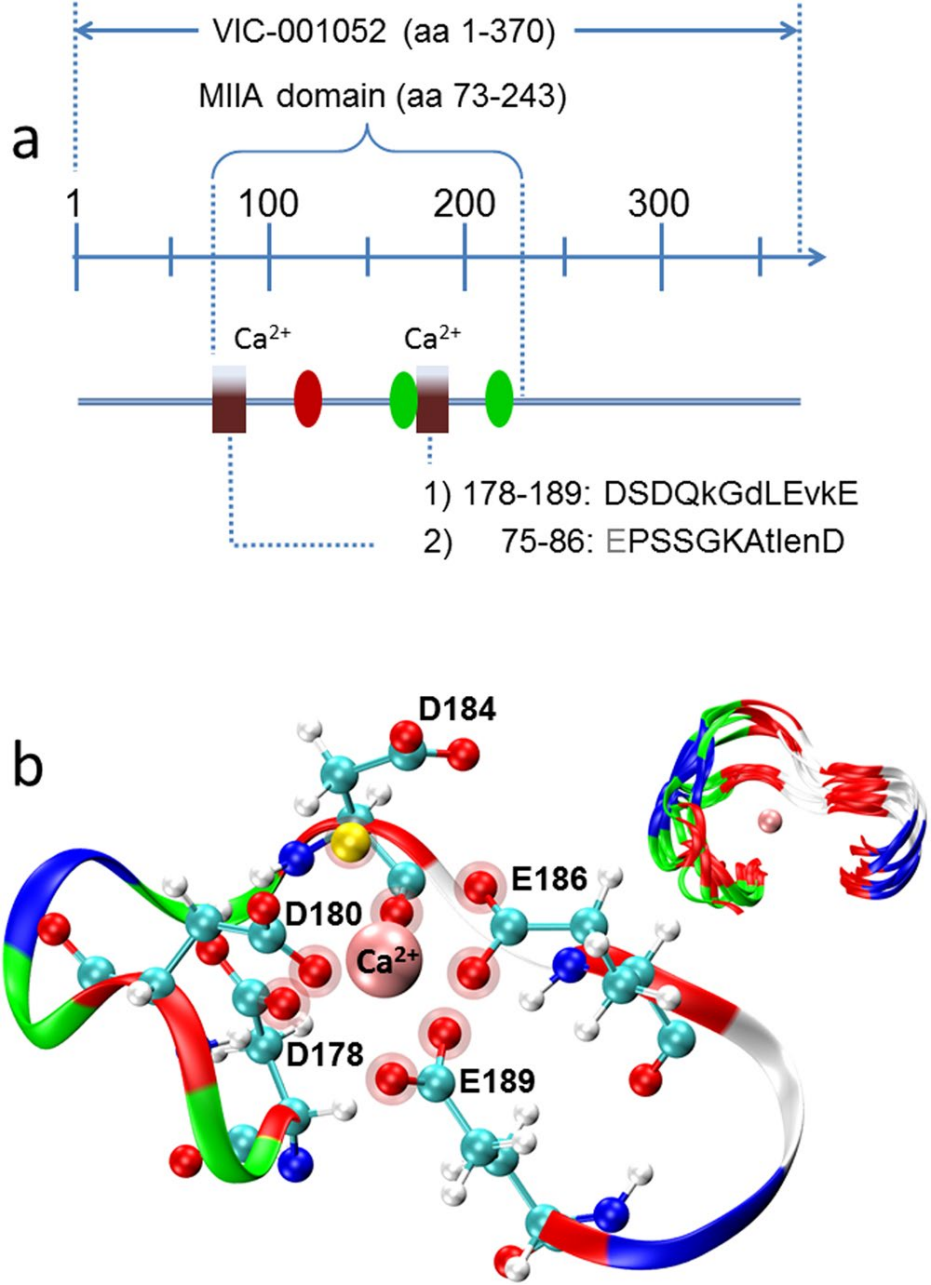
Sequences in the MIIA-domain with similarity to canonical Ca^2+^-binding loops. (**a**) Sequences (Table S1) and their position (boxes) within the primary structure of the MIIA-domain. The gray letter indicates a conservative amino acid replacement with respect to human calmodulin 1; tryptophan residues 172 and 223 (green ovals), GDPH cleavage site (red oval). Capital letters indicate agreement with amino acids at these positions in Ca^2+^-binding loops of EF-hands (gray: conservative replacements). (**b**) Hypothetic structural model of Ca^2+^ coordination by site 1 of the MIIA domain (yellow sphere: a single Ca^2+^-coordinating water molecule). The inset shows the superposition of 10 snapshots (at 40 ns) of the backbone dynamics with largest fluctuations occurring at position 184.

The above spectroscopic data show that the 25 C-terminal amino acids are not essential for folding but do regulate the efficiency of metal-ion-induced structure formation and catalysis. Since Ca^2+^ was still able to support the typical spectral phenotype of the MIIA domain, it is unlikely that a Ca^2+^ binding site is present in the 25 C-terminal residues. The stoichiometry of only two cations binding to the MIIA domain despite the large excess of a negative charge hints at the presence of specific binding site structures, rather than non-specific electrostatic association of the investigated cations with disordered protein regions. The MIIA domain does not harbor a canonical Ca^2+^-binding motif. However, less restrictive sequences derived from the original 12 amino acid-long EF-hand Ca^2+^-binding loop (PROSITE pattern PS00018, Supplemental, Table S2) identified regions whose topology and sequences are displayed in Fig. 6(a). Lifting restrictions exclusively at the fifth position in the 12 residue-long consensus sequence identified a single region with similarity to a Ca^2+^-binding loop (pattern 1 in Fig. 6a). One more region (pattern 2) was detected when additional variability was allowed (Table S2). The sequences complied with eight of the total of ten amino acid positions that specify a canonical Ca^2+^-binding loop (when the conservative D to E replacements is considered consistent with an EF-hand).

Due to the absence of experimentally determined structures of the MIIA domain or the related NopE proteins, we used MD simulations to investigate whether a local Ca^2+^-dependent folding potential may exist within the MIIA domain. Sequences with similarity to canonical Ca^2+^-binding loops are reasonable candidates for such a study. However, the simulations did not intend to prove or disprove binding at specific sites. Instead, we aimed at revealing the potential of short anionic stretches of amino acids in the wt-MIIA domain to reduce backbone flexibility upon Ca^2+^-coordination. The simulations were performed with a structure that was generated by homology modeling of the putative Ca^2+^-binding site 1 of the MIIA domain with human calmodulin EF-hand 3. MD simulations were restricted to the sixteen residues 176 to 191 (MIIA-domain numbering). Among these, the 178–189 segment exhibits the highest similarity to a canonical twelve amino acid-long Ca^2+^-binding loop. During 400 ns of total simulation time, Ca^2+^ stayed bound to the side chain carboxyls of four residues, namely D178, D180, E186, and E189 fluctuating between monodentate and bidentate coordination with Ca^2+^ (Fig. 6b). At the same positions, carboxyl-carrying residues are also found in EF-hands. At the beginning and during the final half of the simulation time, Ca^2+^ was additionally bound to the backbone carbonyl oxygen of D184 in agreement with backbone carbonyl oxygen coordination at the corresponding position in the crystal structure of the template. The high backbone flexibility at this site (Fig. 6b, inset) resulted from a switch between Ca^2+^ coordination via the backbone carbonyl oxygen or the side chain carboxyl of D184. In addition, a water bound to Ca^2+^, leading to a total of seven oxygens in the coordination sphere of Ca^2+^ within less than 2.5 Å as in the EF-hand Ca^2+^-binding loops^23^. Likewise, only the carboxyl at position 12 (here E189) exhibited a stable bidentate metal coordination as is typical of EF-hands.

## Discussion

Host infection by Gram-negative bacteria *via* the type III secretion system (T3SS) requires complete unfolding of the secreted proteins. Intrinsic disorder is over-represented in pathogenic proteins and may have evolved in favor of secretion efficiency but also as a mechanism to target multiple host proteins and to evade immune defense^24^. However, (partial) folding may be required for biological function such as proteolytic activity of a bacterial pathogen in the host. Here, we have discovered intrinsic disorder in the ion-induced auto cleavage domain (MIIA) of the coral pathogen protein VIC_001052 from *Vibrio coralliilyticus*. The location of the VIC_protein in a cluster that contains a T3SS and the sequence similarity of the wt-MIIA domain with regions of the T3SS-secreted NopE proteins of *Rhizobium sp*. suggests that the VIC_protein is secreted in the same way. On a molecular level, it may act as a pathogenic protein in the coral cell or its endosymbiont by coupling metal-ion-binding structural transitions in a similar manner as the NopE proteins which are involved in symbiosis. However, the low secondary structure content of the MIIA domain was not expected, because the previously demonstrated autocatalytic activity implies structure at the catalytic and most likely also at the substrate site (a GDPH motif that is conserved also among NopE proteins). Thus, the MIIA domain features properties that are increasingly recognized for intrinsically disordered regions in eukaryotic proteins that are involved in signaling, where structural substates are populated in diverse ligand protein interactions^25^ or upon cation binding^26^. Here, we have shown that the apparently conflicting features of disorder and catalytic activity can be reconciled by the Ca^2+^-induced folding of the MIIA domain. This differs from the more common role of Ca^2+^ as an allosteric regulator that repositions preformed secondary structure elements in Ca^2+^-regulated proteins, thereby promoting activity by more subtle tertiary structural changes^27^.

We have shown that Ca^2+^ ions bind to the MIIA domain with enthalpies of ~−55 kJ mol^−1^ and a macroscopic K_d_ in the high nanomolar to low micromolar range. In contrast to canonical EF-hands, where Ca^2+^ binding is driven by entropy with only small enthalpic contributions^28–30^, the Ca^2+^-induced secondary structure formation in the MIIA domain is accompanied by an entropy decrease. The gain of favorable interactions within the metal ion-induced folded state contributes to an overall enthalpy-driven and thus exothermic reaction. The thermal stability of the folded state of the MIIA domain is remarkably high and similar to that of the partially urea-destabilized 79 amino acids long N-terminal calmodulin domain (N-Cam) in the presence of 100–200 µM Ca^2+^ ^29^. The binding enthalpy of about −110 kJ for the two Ca^2+^ ions implies an unfolding enthalpy greater than +110 kJ. This agrees with the estimated value of 153 kJ from thermal unfolding which further indicates the additional thermal unfolding of residual structure outside the Ca^2+^-binding sites. However, the electrostatic interactions that underlie ion binding must be rather site-specific, because Ca^2+^ and Eu^3+^ bound with the same 2:1 stoichiometry despite their different charges. Eu^3+^ and Tb^3+^ also evoked secondary structural changes similar to Ca^2+^. Therefore, we believe that the lanthanides occupy the same two sites in the wt-MIIA domain as Ca^2+^ in analogy to proteins for which lanthanides have been used to probe Ca^2+^ binding sites^20–22^. It is remarkable that, despite the low structure-forming propensity of the MIIA domain, ion selectivity for Ca^2+^ and Mn^2+^ is clearly observed in both secondary structure formation and catalytic activity. The coupling of metal ion binding to folding in the MIIA domain may be responsible for efficient ion-discrimination of the MIIA domain as compared to the more subtle ion-specific structural differences in canonical EF-hand proteins. For example, Mg^2+^ recognizes and stabilizes the EF-hand in the N-terminal domain of human calmodulin (N-CaM), a process which has been proposed to confer a regulatory role to Mg^2+^ in Ca^2+^signaling^29^. In the case of the MIIA domain, Mg^2+^ does not stabilize any secondary structure. Nevertheless, there are intriguing similarities such as the interaction of the MIIA domain with Mn^2+^ which also binds to EF-hands^29^.

The response of the wt-MIIA domain to Ca^2+^ is similar to that of the intrinsically disordered protein prothymosin, an unstructured 110 amino acid-long protein that adopts secondary structure and undergoes compaction in the presence of Zn^2+^ but not Ca^2+^ or Mn^2+^ ^31^. Whereas prothymosin is entirely devoid of hydrophobic residues, the wt-MIIA domain folds in accordance with a hydrophobic collapse that leads to reduced solvent accessibility of two tryptophan residues. A similar Ca^2+^-induced lowering of the accessibility of hydrophobic residues has also been reported for the *Nereis* sarcoplasmic Ca^2+^-binding protein (NSCP)^17^, a non-canonical EF-hand protein with larger structural flexibility than calmodulins but lower intrinsic disorder than the MIIA domain. The hydrophobic clustering upon ion-induced folding of the wt-MIIA domain is probably crucial in preserving ion-regulated function This may explain the appearance of Zn^2+^-induced autocatalysis in the W223F and W172F mutants after prolonged incubation with the cation (compare Figs S5 and S7).Finally, catalytic responsiveness to Ca^2+^, Mn^2+^ and Zn^2+^ was strongly suppressed in the C-terminal truncation mutants although they bound these cations and partially adopted secondary structure.

The proposed hydrophobicity-based structure formation in the Ca^2+^-bound state of the VIC_protein may be shared among other MIIA-domain-carrying proteins as shown in the alignment in Fig. S8. Non-polar residues exhibit a high degree of positional conservation which supports a similar fold upon hydrophobic clustering. The molecular mechanism by which this clustering is controlled by ions appears to originate in the interspersed hydrophilic and predominantly negatively charged amino acids. Cation binding would reduce the polarity of these interspersed sequences and thereby enable the formation of more extended stretches with lower charge density in which folding and compaction is no longer prohibited by electrostatic repulsion. The folding-dependent Ca^2+^ regulation of the MIIA domain raises the question which sequence motifs are involved in Ca^2+^ binding and how pathogenicity is linked to Ca^2+^ signaling. Importantly, only the catalysis-stimulating cations were able to also induce secondary structure, demonstrating a tight linkage between secondary structure and biological function despite the intrinsically low structure content of the metal-free wt-MIIA domain. Metal-dependent structure formation included the increase of the CD-signature of turns which are the expected structural feature of Ca^2+^-binding loops. Thus, we addressed the potential of short sequences to form stable loops around Ca^2+^ despite the low secondary structure-forming propensity of the MIIA domain. We have searched putative Ca^2+^-binding sites by similarity to Ca^2+^-binding loops of EF-hands and by vicinity to residue W172 where a strong metal-dependent increase of hydrophobicity (loss in local dielectric constant) was observed. Both criteria identified residues 178–189 where charge neutralization would reduce hydrophilicity near W172 and indirectly by loop formation which could bring W172 in the vicinity of conserved non-polar residues on the C-terminal side of E189 (Fig. S8). Our MD simulations do not prove but support the possibility of Ca^2+^-binding loop formation in the MIIA-domain. Although the negative charge of D184 within the proposed 12 amino acid-long metal-binding sequence does not comply with the canonical of EF-hand pattern, it did not cause dissociation of the complex in the simulations but exhibited large local structural fluctuations (coordinating Ca^2+^ either *via* its side chain or the backbone carbonyl oxygen). This behavior may reflect a fundamental molecular property of the pathogenic or symbiosis-mediating proteins. They may harbor “close to canonical” Ca^2+^-binding motifs which are destabilized by the replacement of structure-promoting residues by amino acids that favor disorder. Thereby, only the additional cation binding energy promotes structural stability in these regions which is in line with recent views of the role of protein disorder in structure function relationships^32^. The MD result shows that such a replacement could well preserve cation binding but at the same time it would enhance translocation efficiency across membranes by lowering the unfolding energy.

## Conclusions

**Figure 7:**
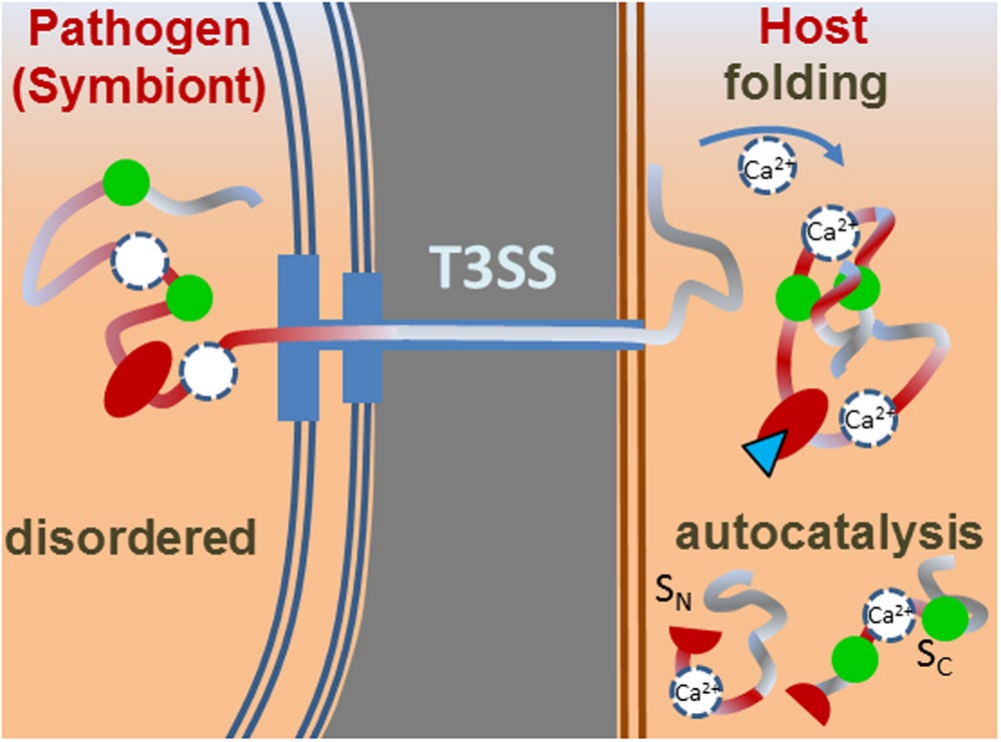
Schematic representation of the secretion of the unfolded MIIA domain and its Ca-dependent folding and catalytic activity in the host cell. Two Ca^2+^-binding sites (white filled circles) detected in this study lie at yet unidentified positions. At low Ca^2+^ concentration in the pathogen, the binding sites are unoccupied and the MIIA domain unstructured for efficient secretion by T3SS. At elevated Ca^2+^ concentrations in the host, two Ca^2+^ ions bind to the MIIA domain (dissociation constant K_d_ < 1.6 µM) and induce hydrophobic collapse with clustering of intrinsic tryptophan residues (green filled circles) into the interior of the folded protein. Thereby, autocatalytic activity cleaves the GDPH site (red filled oval) leading to release of the fragments S_C_ and S_N_ which promote biological function.

Irrespective of the yet ambiguous assignment of the two metal-binding sites, the biological function of Ca^2+^-induced secondary structure formation in the MIIA domain is likely to provide a sensor/signaling mechanism during early infection. The high sequence similarity with NopE proteins suggests that a common molecular “post secretion host-sensing” conformational switch may exist in both beneficial and pathogenic T3SS-dependently secreted proteins. Ca^2+^-dependent disorder-to-order transitions are typical of numerous proteins secreted by type 1 secretion systems^33,34^. It is thus remarkable that the MIIA domain of the coral pathogen resides in a T3SS gene cluster, but exhibits a similar Ca^2+^-dependent transition from a disordered secretion-adapted state to a more compact structure with hydrophobic interactions. This may provide the molecular mechanism for triggering enzyme activity and effector recognition under the influence of Ca^2+^ signals in the host(s) as sketched in Fig. 7.

## Materials and Methods

### Construction of plasmid pVCD020

Plasmids and oligonucleotides used in this study are listed in Table S1.

Plasmids were propagated in *Escherichia coli* DH10B (Thermo Scientific). All plasmids derived from pVCD019^35^, which encodes a fusion protein consisting of MalE, the MIIA domain of the hypothetical protein VIC_001052 (amino acids 73–243 of the full-length protein) and a Strep-tag (Table S1). Derivatives were generated by applying the QuikChange protocol (Agilent Technologies) with the primer pairs listed in Table S1. The integrity of the modified MIIA domain was confirmed by nucleotide sequencing. For fluorescence spectroscopy of single intrinsic tryptophans, the MIIA domain was purified by a FLAG-tag, because this tag does not contain additional tryptophan residues.

### Protein expression and purification

For protein purification, the corresponding plasmids (Table S1) were transformed into *E. coli* BL21 (DE3) (NEB). Cells were grown in lysogeny broth containing ampicillin (150 µg/ml) at 37 °C. At an OD_600_ of about 0.7, protein expression was induced by Isopropyl-β-D-thiogalactopyranosid (IPTG, 200 µM final concentration) and cells were grown at 28 °C for 4 h. Cells were harvested and washed once with TKE buffer (100 mM Tris, 200 mM KCl, 10 mM Ethylendiamintetraacetat (EDTA), pH 8,0). Cells were resuspended in TKE buffer containing lysozyme (0.1% w/v) and incubated for 1 h on ice. Cells were disrupted by sonication. Cell debris was removed by centrifugation and a final filtration step. The fusion protein was purified by affinity chromatography using a MBPTrap^TM^ HP column (GE Healthcare) and eluted in TKE buffer containing 10 mM maltose. The MalE tag was removed by cleavage with thrombin over-night at 22 °C (GE Healthcare). The Strep-tag-carrying MIIA domain was purified using a StrepTrap^TM^ HP column (GE Healthcare) and eluted in TNE buffer (100 mM Tris-HCl, 150 mM NaCl, 1 mM EDTA, 2.5 mM desthiobiotin, pH 7.4). Anti-DYKDDDDK (FLAG®) G1 Affinity Resin; GenScript (Piscataway Township, USA) was used for purification of FLAG-tagged proteins according to the manufacturer’s protocol. For concentration of the protein and changes of the buffer, Amicon^®^ Ultra Centrifugal filters (Ultra-15 10 K, Merck) and Slide-A-Lyzer^®^ Mini dialysis devices (3.5 K molecular weight cutoff, Thermo Scientific) were used. Protein samples were analyzed by SDS-PAGE^36^. Gels were stained with Coomassie Brilliant Blue G-250.

### Metal ion-induced autocleavage

5 µg of protein (MIIA domain) in a volume of 10 µl were incubated for 30 min at room temperature with different metal salts (Ca, Mg, Mn, Eu, Tb) in Tris buffer (100 mM Tris-SO_4_, pH 8.0). The reaction was stopped with 10 mM EDTA and analyzed by SDS-PAGE.. For negative control, the metal ion solutions were replaced by Tris buffer. In addition to these standard conditions (Fig. S7)^5^, SDS-PAGE was performed after completion of spectroscopic data acquisition which entailed longer incubation times of 2 hrs up to overnight storage of the spectroscopy samples at 6 °C and required a lower concentration of 2 mM Tris to avoid strong UV-absorption.

### Circular dichroism (CD)

Circular dichroism (CD) spectra were recorded with a J-815 spectrometer (JASCO, Gross-Umstadt, Germany) equipped with a continuously stirred temperature-controlled 1 cm cuvette. For thermal denaturation, the temperature increased at a rate of 1 °C/min from 18 to 92 °C. Two spectra were then accumulated between 185 and 250 nm (5 nm band width, 120 nm/min) at constant temperature in intervals of 2 °C. Spectra were analyzed by Dichroweb^37,38^ using the method CDSSTR (data set 6). Thermal unfolding data were analyzed by a two-state unfolding model. The temperature-dependent mol-fraction f_N_ of the native state was expressed as f_N_ (T) = (1 + exp(ΔS·T-ΔH)/R·T)^−1^. The unfolding enthalpy ΔH and entropy ΔS were determined from this with the additional condition ΔH/ΔS = T_m_, where T_m_ is the experimentally determined midpoint temperature of 50% denaturation.

### Fluorescence spectroscopy

Intrinsic tryptophan fluorescence was measured at room temperature in a 100 µL cuvette using an LS55 spectrometer (PerkinElmer Life Sciences) with excitation at 285 nm and emission recorded from 290 to 450 nm (band width of 5 nm).

### Isothermal titration calorimetry (ITC)

Binding isotherms were measured with a MicroCal VP-ITC Instrument (Malvern Instruments, Herrenberg, Germany). After an initial injection of 5 µL of CaCl_2_ (2 mM), 10 µL aliquots were further injected to 1.4 ml of MIIA-D116A domain (55 µM). Both solutions contained 20 mM Tris-SO_4_ at pH 8. The data were processed and analyzed using the MicroCal origin 7.5 software.

### Mass spectrometry

Mass spectrometry measurements were performed by using nano-ESI-FTMS. Samples were injected into nanospray capillaries from New Objective Inc. (Woburn, MA, USA) with an inner tip diameter of 2 mm and analyzed with a Velos Pro Orbitrap Elite mass spectrometer (Thermo Fisher Scientific Inc.,Waltham, MA, USA). The temperature of the transfer capillary was fixed at 240 °C. All measurements were performed in the positive ionization mode with an applied voltage to the nanospray capillary of 3 kV. The voltages applied to the S-Lens and multipoles were automatically tuned to favour the transmission of high m/z values. Full MS spectra were recorded using the Orbitrap analyzer, by averaging at least 110 scans in the range of m/z 2000–4000. Acquisition and treatment of the data was done with the Xcalibur software.

### Molecular dynamics simulations

Molecular dynamics (MD) simulations and data analyses were performed using the AMBER 14 program package with the ff99SB force field applied to the protein domain^39^. Additional parameters were employed for the bound Ca^2+^ ion^40^. The starting structure of a hypothetical Ca^2+^-binding site was generated by homology modelling using “SwissModel”^41^ with a 30 amino acid-long target sequence of the MIIA domain that contained the 12 amino acid-long subsequence (amino acid 178 to 189) which exhibited the highest similarity to the sequence pattern of Ca^2+^-binding loops in canonical EF-hands (PROSITE pattern PS00018). The Ca^2+^-binding loop of EF-hand 3 of human calmodulin (UniProtKB - P0DP24, CALM2_HUMAN) in the NMR structure (pdb-file 2kuh) served as the template;

EF-hand 3 Ca^2+^-binding loop: FDKDGNGYISAAELRH,

MIIA-fragment: Y^173^IEGIDSDQKGDLEVKEYNNINYSGGTVND^202^.

MD simulations were carried out with the modelled structure of amino acid 178 to 189 (underlined above) to which a single Ca^2+^ ion was added at the position corresponding to that in the Ca^2+^-binding loop of calmodulin. The protonation state of the domain was adjusted to mimic the physiological pH. TIP3P waters were added with a minimum water layer thickness of 12 Å. Five hundred steps of steepest decent and five hundred steps of conjugate gradient with 500 kcal mol^−1^ Å^−1^ harmonic constraint on the protein were initially conducted after which another one thousand steps of steepest decent and 1500 steps of conjugate gradient were performed without constraints. The system was heated from 0 to 300 K with 10 kcal mol^−1^ Å^−1^. A harmonic constraint on the domain was performed for 40 ps followed by another 2 ns preconditioning run at 300 K without constraining the solute. Finally, a 400 ns MD run was performed with periodic boundary condition in the NPT ensemble. Simulations were terminated and restarted every 50 ns. The SHAKE algorithm, a 2 fs time integration step, 10 Å cutoff for non-bonded interactions and the particle mesh Ewald (PME) method were used.

## Supplementary information

Supplementary Information
