# Supplementary material for: Calcium binding to a disordered domain of a type III-secreted protein from a coral pathogen promotes secondary structure formation and catalytic activity
Source: Sci Rep. 2019 May 8;9:7115. doi: 10.1038/s41598-019-42898-0 (PMC6506597; doi:10.1038/s41598-019-42898-0)
Supplement: Supplementary file 1 — Supplementary Information [file 41598_2019_42898_MOESM1_ESM.pdf]

Supplementary Information

Calcium binding to a disordered domain of a type III-secreted protein from a coral pathogen promotes secondary structure formation and catalytic activity

Elisabeth Hoyer[2†], Julius Knöppel[1], Martina Liebmann[2], Michael Steppert[3], Manuel Raiwa[3], Olivia Herczynski[2], Erik Hanspach[2], Susanne Zehner[2], Michael Göttfert[2], Satoru Tsushima[1], Karim Fahmy[1], Jana Oertel*[1]

[1] Helmholtz-Zentrum Dresden – Rossendorf, Bautzner Landstrasse 400, D-01328 Dresden, Germany

[2]Technische Universität Dresden, Institute of Genetics, Zellescher Weg 20b, D-01217 Dresden, Germany

[3]Leibniz University Hannover, Institute of Radioecology and Radiation Protection, Herrenhäuser Str. 2, D-30419 Hannover, Germany

† present address: Ludwig-Maximilians-Universität München, Department of Biology I, Microbiology, Großhaderner Str. 2, D-82152 Planegg-Martinsried, Germany

*Corresponding author:

Jana Oertel

Helmholtz-Zentrum Dresden – Rossendorf, Bautzner Landstrasse 400, D-01328 Dresden, Germany

e-mail: j.oertel@hzdr.de

1. Disorder prediction in the MIIA domain

Figure S1. Amino acid sequences of the hypothetical protein Vic_001052 and two nodulation proteins exhibiting similar MIIA domains. **(a)** Amino acid sequence of the hypothetical protein Vic_001052 of *Vibrio coralliilyticus* ATCC BAA-450 (accession number: EEX34258), the analyzed MIIA domain is shaded in gray. The aspartate residue at the cleavage site is indicated in bold and underlined. **(b)** Amino acid sequence of NopE1 of Bradyrhizobium japonicum USDA110 (accession: WP_011084615). **(c)** Amino acid sequence of NopE2 of *Bradyrhizobium japonicum* USDA110 (accession:WP_011084464).
The two MIIA domains in each of the NopE proteins are shaded in gray and the two aspartate residues at the cleavage sites are indicated in bold and underlined. Disordered regions (red) and "unfoldability" are based on averaged absolute net charge and hydrophobicity as predicted by FoldIndex©[1] with window size 51 (https://fold.weizmann.ac.il/fldbin/findex) and PONDR®[2] using the VL3-BA predictor (http://www.pondr.com/).

a

MIMAEINSFQISWSVNTQSHQHQSNNTEMHRNQVSRGGPNTQTNSSNDTTSSNGSSWGGPVE
PDDGSSYDLKSYEPSSGKATLENDRYTINIDESSSEIEVIDKQNPEDSFRIYG**D**PHFDIGNDGDT
DFDFKKDMSIELDDGTKLHIHTTPTSNGETLATSLAIEEPDGSGWYIEGIDSDQKGDLEVKEYN
NINYSGGTVNDDAALELQVRDGNYFLNSDNGWETLEEDKKASNDAIVNDIETAIKNQPWGIS
NQEDFLNDMYANNPNSRTFEDYLSSVKEMFEQMGITSNNVNATEPDEKSDREKVLDALSLLS
QLYNTLNNMNDRINNPNQTNLGDSIKNVHSFNNDQLFGQGQNNNRGVVVITIRAA

unfoldability -0.222 (Charge: 0.175, Phobic: 0.397); PONDR: 57.31 % disorder

b

MQYLPVAGLPVVGAPDSMNGVAPEGAVVTPTFNAMLGQYAPASYQYLPVASPALVGTAVG
SVVPVVVAPAVVTTQAYMMAPPPSTMLMSQLNQNAEPPVDPVWTHEVKDGKATINLGDKY
TITANEKDGTWTVRNNQTGHVTVIHG**D**PHVDADGDGKDDFDFKKDMTFQLDDGTKITVNN
VDYGNGQAISSKLTITNGHNAIVVEGLGDDKDGKNNLRVTQSNAGMTLDELTSDGAQTIHES
GQGWVDGAGRAVNQASIDDGEQGRGPGNYQYASGAPTSTAPVFFVPPPPPPLAMVPVAAAQ
VPTQSSQPAPVWSHEVRDGKAEIKLGDKYSILVDENDGTVLIRNSQTGKITSIKG**D**PHVDADG
DGKVDFDFKKNMTFQLDDGTKITVDTVDIGKGKTMASKLTITNGDNAMVVEGLGDRFDGK
NNLKVTQSNAGRTLDQLTSDGAQTIYEQPGSGWVDRSGRQVNQEIIDSNENPGTTSDA

1st domain: unfoldability -0.103 (Charge: 0.076, Phobic: 0.404); PONDR: 28.82 % disorder
2nd domain: unfoldability -0.047 (Charge: 0.053, Phobic: 0.415); PONDR: 34.12 % disorder

c

MAYLPVAGLPVVGALDSTVNGVGSEGVVASPAFSTLLGQYVTPGAYQYLPAGAAVVVAPV
VVPAGGVTTAVVAPAVVTPVVAAPVYMMAPPPPFMLLSQQSSGSEPSVDPVWTHEVKDGKA
TINLGDKYTITANEKDGTWTVRNNQTGHVTKIHG**D**PHVDANGDGKDDFDFKKDMTFQLDD
GTKITVNNVDYGNGETISSRLTITNGHNAMVVEGLGDDKDGKNNLRVTQSNAGMTLDELTP
DGAQTIHESGQGWVDGAGGVVNQASIDAGEQGRAPGNYQSASATPASATPVFFVPPPPPPLA
MVPFAVAQTPAPSSQPAPVWSHEVKDGKATINLGDKYTITADEKDGTWTVRNNQTGHVSRI
HG**D**PHVDANGDGKDDFDFKKGMTFQLDDGTKITVDTVNYGKGKTISSKLTITNGDNAMVVE
GLGDDKDGKNNLKVTQSNAGRTLDQLTSDGAQTIHETNQGWVDNSGRKVTQSVINHNENPD
APPDFKTMMREAMFRRIFGRDAQPA

1st domain: unfoldability -0.098 (Charge: 0.071, Phobic: 0.404); PONDR: 35.29 % disorder
2nd domain: unfoldability -0.108 (Charge: 0.041, Phobic: 0.389); PONDR: 67.65 % disorder

2. Circular Dichroism spectra

Figure S2. Circular Dichroism spectra of the D116A-MIIA domain as a function of metal cation binding. (**a-c**) CD spectra in the absence of metal ions (light gray) and in the presence of Mn^{2+} at 100, 250, and 500 µM (from gray to black), Eu^{3+} (50 mM) and Tb^{3+} (50 µM) . Pie charts represent the relative amount of secondary structure deduced from the data using Dichroweb.[3,4] Spectra were averaged from four consecutive scans. (**d**) Room temperature CD spectra showing the large degree of reversibility of thermal unfolding of the D116A-MIIA domain in the presence of 100 µM Ca^{2+} before (blue) and after heating to 92 °C. Signal to noise is lower because single scans were recorded.

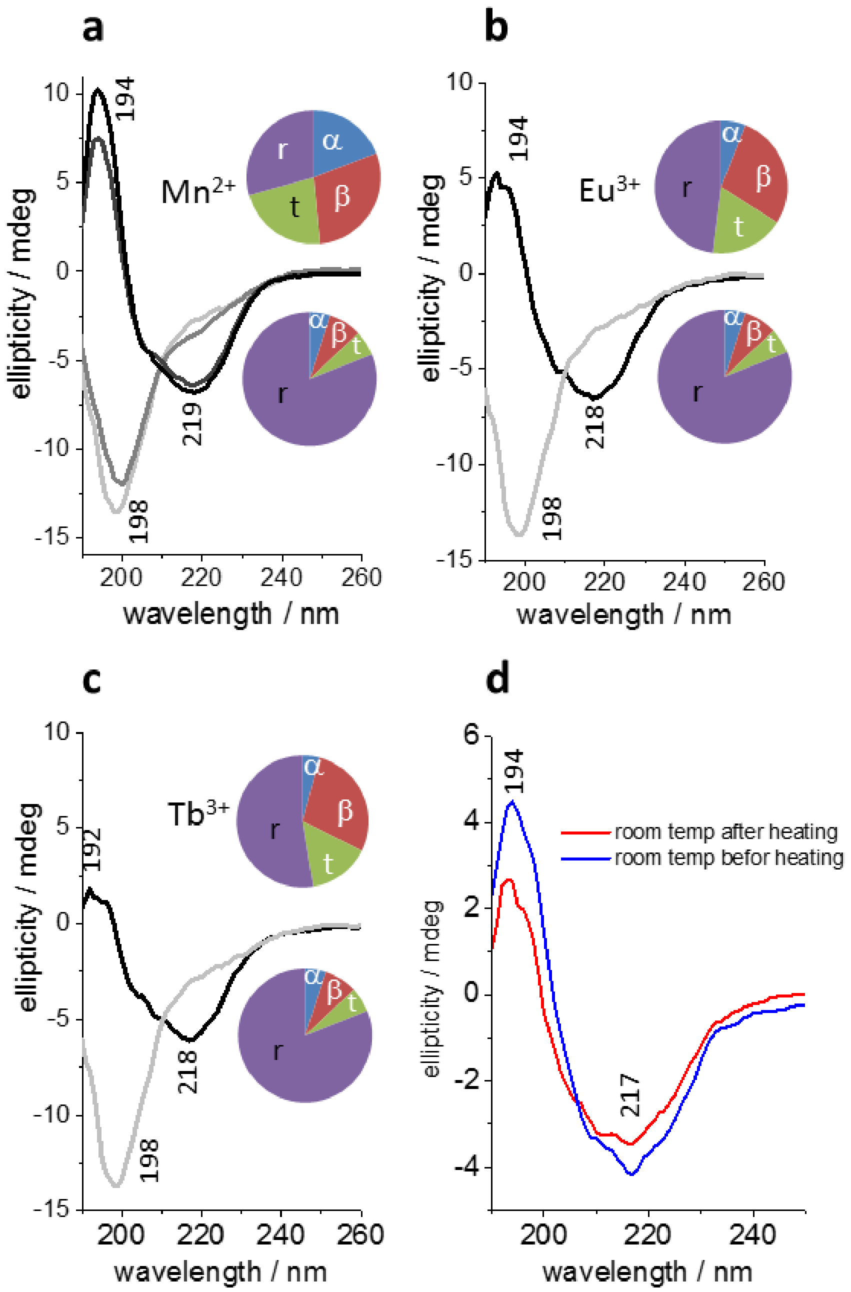

Figure S3. Cation-dependent Circular Dichroism spectra of tryptophan replacement and C-terminally truncated versions of the wt-MIIA-domain. (**a**) W172F-MIIA domain, (**b**) W223F-MIIA domain. Both derivatives were expressed and purified with a FLAG-tag. (**c**) MIIA73-223 lacking 20 C-terminal amino acids of the wt-MIIA domain. (**d**) MIIA73-218 lacking 25 C-terminal amino acids of the wt-MIIA domain. Both truncated derivatives were expressed and purified with a Strep-tag.

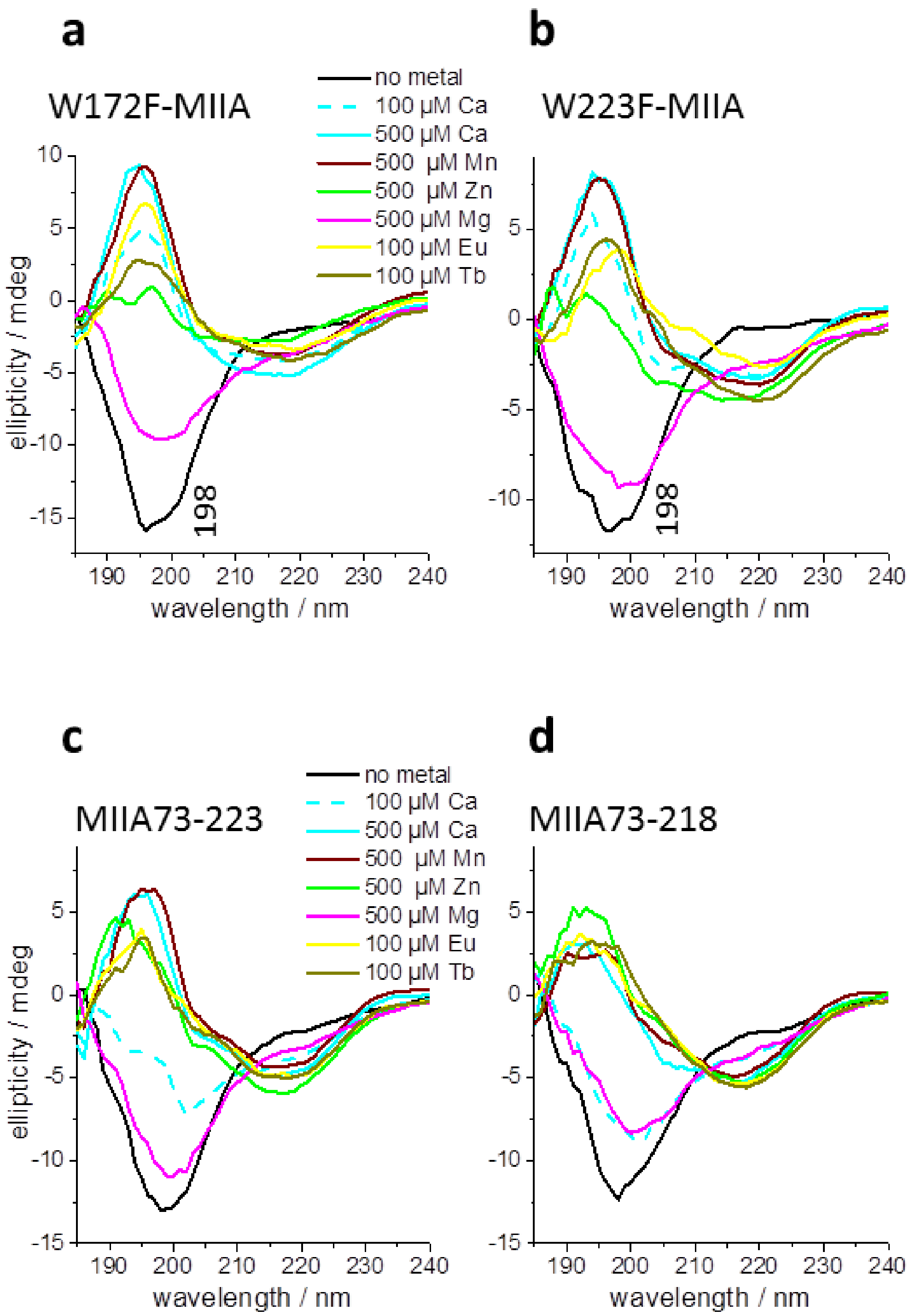

3. SDS-PAGE images

The catalytic activity of MIIA domain constructs was determined by SDS-PAGE. Aliquots where taken from the samples that had previously been investigated spectroscopically at room temperature (Figs. S4 to S6). The additional intermittent storage between spectroscopy and SDS-PAGE (at least overnight storage at 6 °C) allowed partial catalytic cleavage in the presence of Mg^{2+} at concentrations above 200 µM, which does not occur under standard conditions (30 min at 25 mM cation concentration)[5] as also shown here (Fig. S7).

Figure S4. Coomassie-stained SDS-PAGE of wt-MIIA domain after metal ion-induced autocatalytic cleavage**.** (**a**) wt-MIIA domain expressed and purified with a Strep-tag (1.8 µg protein loaded per lane), (**b**) wt-MIIA domain expressed and purified with a FLAG-tag (2 µg protein loaded per lane). The type of cation and its concentration (µM) for catalytic activation is shown above each lane of the gels. Molecular weight standards are indicated on the left. M_F: full length wt-MIIA domain, S_C: C-terminal cleavage product, S_N: N-terminal cleavage product.

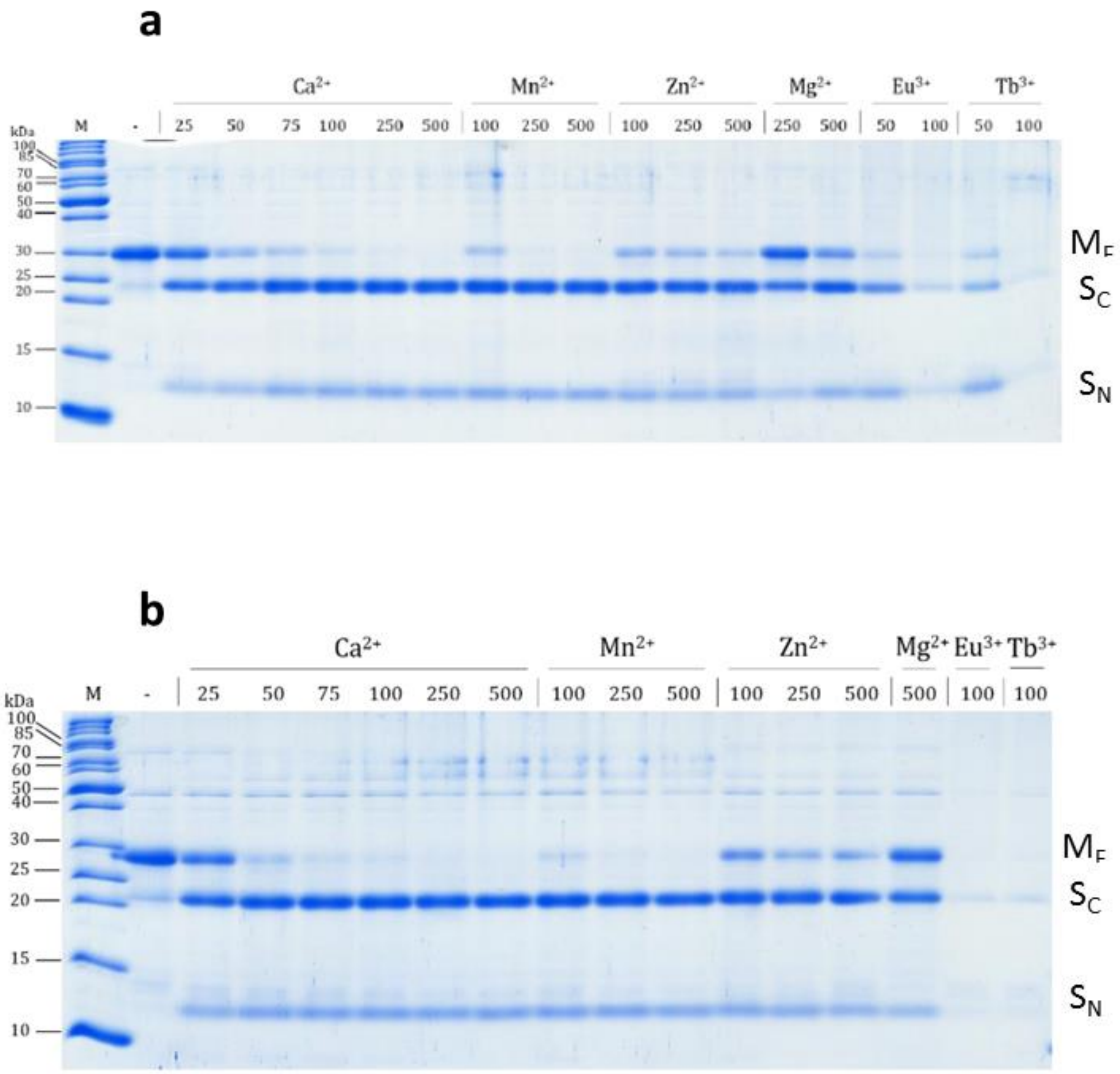

Figure S5. Coomassie-stained SDS-PAGE of tryptophan replacement variants of the MIIA domain after metal ion-induced autocatalytic cleavage. (**a**) W172F-MIIA domain (**b**) W223F-MIIA domain. Both constructs were expressed and purified with a FLAG-tag (2 µg protein loaded per lane). The cations and their concentrations (µM) for catalytic activation are plotted above each lane of the gels. Molecular weight standards are indicated on the left. M_F: full length wt-MIIA domain, S_C: C-terminal cleavage product, S_N: N-terminal cleavage product.

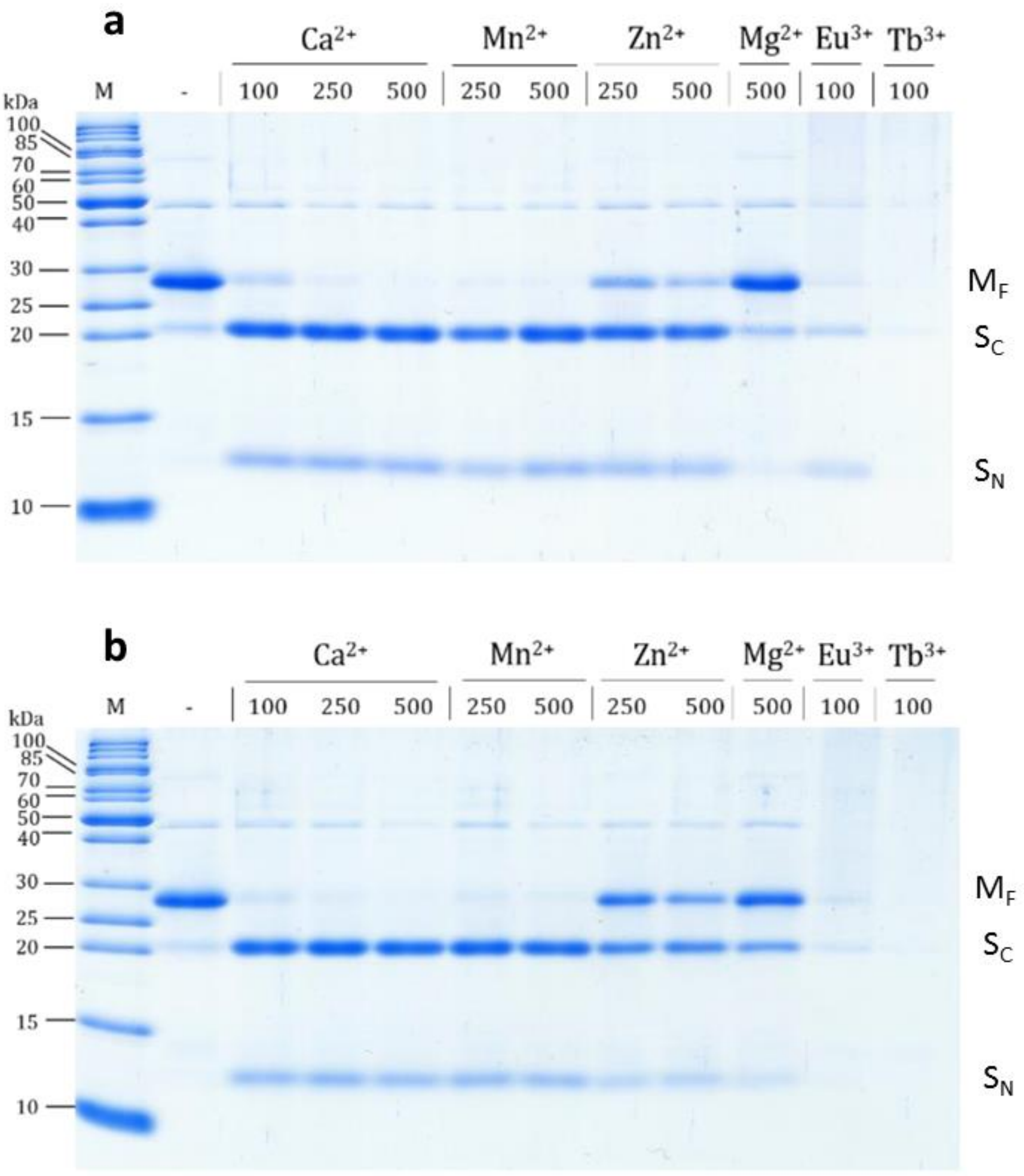

Figure S6. Coomassie-stained SDS-PAGE of C-terminally truncated variants of the MIIA domain after metal ion-induced autocatalytic cleavage**.** (**a**) MIIA73-223 lacking 20 N-terminal amino acids of the wt-MIIA domain (1.8 µg protein loaded per lane), (**b**) MIIA73-218 lacking 25 N-terminal amino acids of the wt-MIIA domain (2 µg protein loaded per lane). The cations and their concentrations (µM) used for catalytic activation are plotted above each lane of the gels. (-) denotes the absence of cations. Molecular weight standards (M) are indicated on the left. M_F: full length wt-MIIA domain, S_C: C-terminal cleavage product, S_N: N-terminal cleavage product. S': unidentified additional cleavage products.

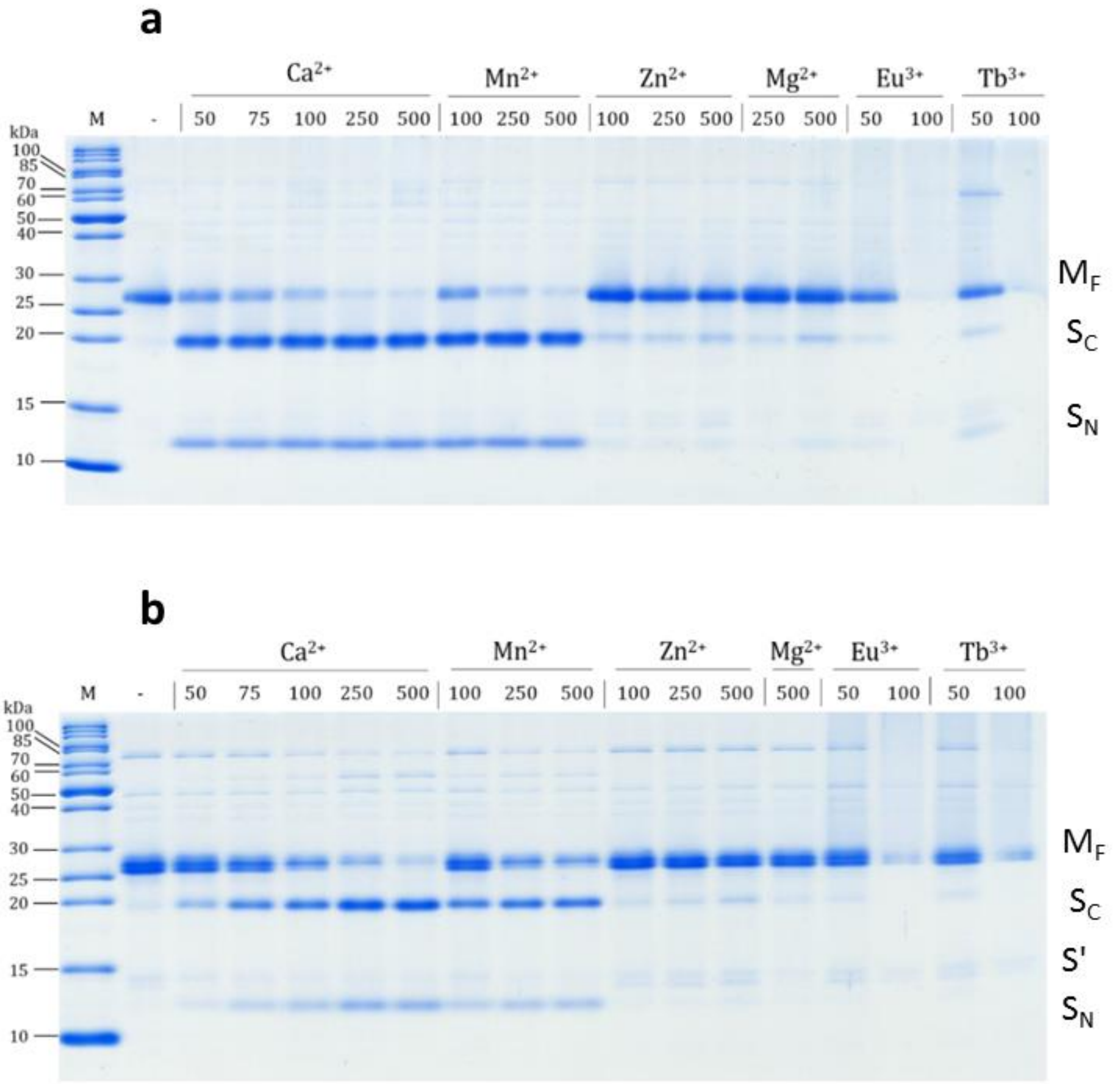

Figure S7: Coomassie-stained SDS-PAGE of the catalytically active constructs of the MIIA domain in the presence of activating and non-activating divalent cations. Catalytic activity was assessed under previously established standard conditions[5] using 30 min incubation with the indicated cations at 25 mM concentration in 50 mM Tris, 200 mM KCl, 10 mM EDTA, pH 8.0. (**a**) Full length and C-terminal truncation mutants of the MIIA domain with Strep-tag. (**b**) Single tryptophan replacement mutants with FLAG-tag, which were used for tryptophan fluorescence measurements.

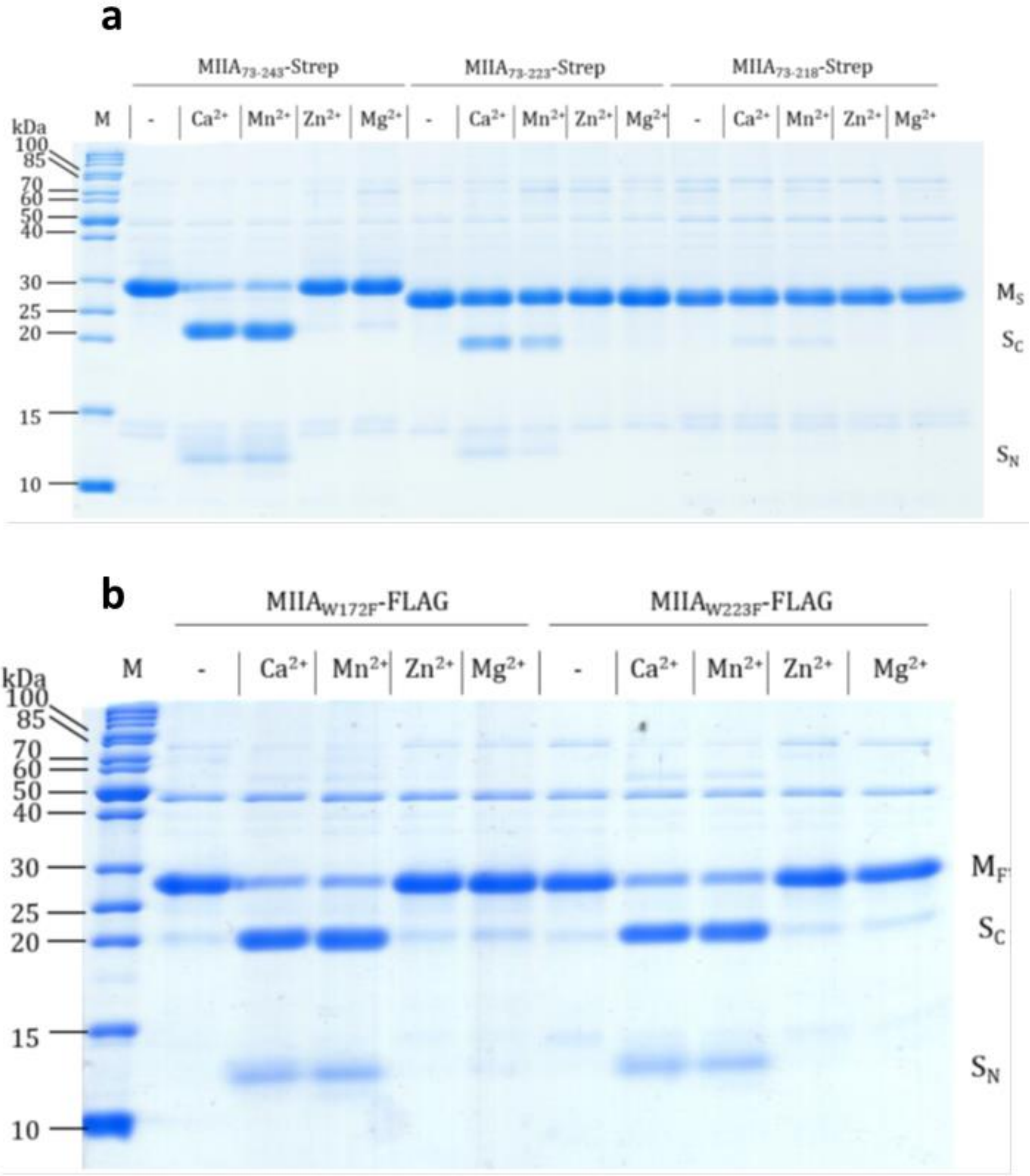

4. Sequence alignments

Figure S8. Multiple alignment of the MIIA domain of *Vibrio coralliilyticus* (WP_006958402, now EEX34258) with selected MIIA domain sequences from different Proteobacteria. **(a)** Highly conserved amino acid positions (>90 %) are marked in red, less conserved ones in blue (≥50 %). **(b)** As in **(a)**, hydrophobic amino acids highlighted in yellow. Purple boxes: hypothetical Ca^{2+}-binding motifs based on their partial similarity to EF-hand Ca^{2+}-binding loops (Table S2). Turquoise box: 25 amino acid-long C-terminal truncation of the MIIA-domain in the MIIA73-218 mutant. Tryptophan residues of the wt-MIIA domain are labeled by residue number. The *Bradyrhizobium* strains encode proteins with two MIIA domains, indicated as N-terminal (N) and C-terminal **(c)**. Homologous proteins are frequently found in *Bradyrhizobium* strains (data not shown). The alignment was done using multalin.[6] WP_011084615, *Bradyrhizobium japonicum* USDA 110 (NopE1); WP_011084464, *B. japonicum* USDA 110 (NopE2); WP_057861928, *B. lablabi* CCBAU 23086; WP_057850284, *B. valentinum* LmjM3; OHV14582, *Methylobacterium extorquens* CP3; WP_007462645, *Photobacterium marinum* AK15; WP_081833258, *Paraburkholderia fungorum* NBRC 102489; ACD19565, *Paraburkholderia phytofirmans* PsJN; WP_051963486, *Caulobacter henricii* CF287; WP_056613648, *Sphingomonas* sp. Root241; ODB99281, *Candidatus* Thiodiazotropha endoloripes isolate="G_E" ; WP_082532857, *Pelomonas* sp. Root1237; WP_090449629, *Dyella* sp. OK004. Multiple sequence alignment with hierarchical clustering was used[6].

a

```
                   73                                             116
WP_006958402        SYE-PSSGK ATLE-NDRYT INIDESSSEI EVIDKQNPED SFRIYGDPHF DI--GNDGDT DFDFKKDMSI ELDDGTKLHI HTTPT-S-NG
WP_011084615 N     WTHE-VKDGK ATINLGDKYT ITANEKDGTW TVRNNQTGHV T-VIHGDPHV DA--DGDGKD DFDFKKDMTF QLDDGTKITV NNVDY-G-NG
WP_011084464 N     WTHE-VKDGK ATINLGDKYT ITANEKDGTW TVRNNQTGHV T-KIHGDPHV DA--NGDGKD DFDFKKDMTF QLDDGTKITV NNVDY-G-NG
WP_057850284 N     WTHE-VHDGK AAIHLGDKYT ITADEKDGTW TVRNNETGHV T-KIHGDPHF DA--DGDGKD DFDFKKGMTL KLDDGTKITV DTADY-G-NG
WP_011084464 C     WSHE-VKDGK ATINLGDKYT ITADEKDGTW TVRNNQTGHV S-RIHGDPHV DA--NGDGKD DFDFKKGMTF QLDDGTKITV DTVNY-G-KG
WP_057850284 C     WSHE-VHDGK ATIHLGDKYT ITADEKDGTW TIRNKETGHV S-KIHGDPHV DA--NGDGKD DFDFKKGMTL QLDDGTKITV DTVDY-G-KG
WP_011084615 C     WSHE-VRDGK AEIKLGDKYS ILVDENDGTV LIRNSQTGKI T-SIKGDPHV DA--DGDGKV DFDFKKNMTF QLDDGTKITV DTVDI-G-KG
    OHV14582       WSHE-VKDGE ATIQLGDKYT VKASEKDATW TVTNNETGNT T-KVSGDPHV DI--DNDGKN DFDFKKDMTF KLDDGTKITV GTVPG-GENG
WP_007462645       WSVE-GNQ-- --IKLDNGYV VTVGNENRDW TITD-AAGNT T-KIWGDPHV DE--NGDG-T DWDFKQNVTF ALDDGTKISV GTAER-G-YA
WP_081833258       WTATPVTNNK ASIDLG-NYK LDFNKSDSSM TMTSKSSGDT T-KIWGDPHL TQHANGANSS TAMFNGPMTF QLPDNTKVTV GTQADKNNKS
    ACD19565       WSNTQVNDNK STIDLG-NYK LDLNKKDSSM LLTDKKSGET T-KVWGDPHI D-----SNGT SNMFNGPLSL NLSDGTKITV GTQGKGN---
WP_051963486       FTAAMPESGK AEIDLGDGNT LSINEASSEV VIRDAD-GNA T-RIWGDPHV SY--NG--KQ VGDFWGTTTF VLENGTKITI NTETSKWN-N
WP_056613648       WTVKTGTEGK ASIDLGDGYS LQLNENNSEM TITNANTGET T-RIWGDPHV DV--NG--QH VYDFWGTTTF TLDNGTKITI NTEQGQGNPN
    ODB99281       WTVCQQENGR ATIDLGDKYE LELNENKSQM IIRNKANGEE T-NIWGDPHV DW--NGDGKT DVDFWGKTTF QLEDGTKITI DTEKWKGNEN
WP_082532857        HASTQMKGG QAVFENDNYR ITAGDNNT-V NIFNKKTGET Y-NIWGDPHV NI--DG--KH AFDFYGTTTF KLEDGTKVTI ETTPWNAGNG
WP_090449629       WNVEANDGQH ARIDLG-HYT LDIDQSSSQF VLTNKATGEV T-RIWGDPHF DN--NG--QA IGTFKGTLTL SLDDGTKLTI NTTP--AGNG

                                     172                                                       223                  243
WP_006958402       ETLATSLAIE EPDGSGWYIE GIDSDQKGDL EVK-EYNNIN YSGGTVNDDA ALELQVRDGN -YFLNSDNGW ETLEEDKKAS NDAIVNDIET
WP_011084615 N     QAISSKLTIT NGHNA-IVVE GLGDDKDGKN NLRVTQSNAG MTLDELTSDG AQTIHES-GQ -GWVDGAGRA VNQASIDDGE QGRGPGNYQ
WP_011084464 N     ETISSRLTIT NGHNA-MVVE GLGDDKDGKN NLRVTQSNAG MTLDELTPDG AQTIHES-GQ -GWVDGAGGV VNQASIDAGE QGRAPGNYQ
WP_057850284 N     KSISSKLTIT NGSNA-MVVE GLGDDKDGAN NLKVTQSNAG LTLDELTADG SQTIHEQ-GQ -GWVDGAGRE VDQASIDAGE EGRAPGAGS
WP_011084464 C     KTISSKLTIT NGDNA-MVVE GLGDDKDGKN NLKVTQSNAG RTLDQLTSDG AQTIHET-NQ -GWVDNSGRK VTQSVINHNE NPDAPPDFK
WP_057850284 C     KTISSKLTIT NGDDA-MVVE GLGDKKDGAN NLKVTQSNAG RTLDQLTADG AQTIYEQ-DQ -GWVDRSGWH VNQASIDANE QAA
WP_011084615 C     KTMASKLTIT NGDNA-MVVE GLGDRFDGKN NLKVTQSNAG RTLDQLTSDG AQTIYEQPGS -GWVDRSGRQ VNQEIIDSNE NPGTTSDA
    OHV14582       TTFSSSLTIT NGDKA-MQVT GLGDSHDGEN NLEVKQSDAG TTLDELENDG SATVYEN-GG -AWQTKDGKA VDQSIIDAAE AAAA
WP_007462645        NTVTDSLTIT KGNQH-IQVT GIADNNPTIS QPKLD----G LEVDAATNDG TVFYANGSTN -SWFTDKESQ NRVTSNQAMG SQQVINEDDT
WP_081833258       VSYADSVTIT HGNDA-YQVT GLSQQNSTGL SVQKSH-D-G RALDAATPDG YTLVANRNGS -GFVDPKTGK QPTEDQIRK
    ACD19565       VSYADKLTIT KGNDA-YLVN GLSEKDSNPL TVQHAG-N-G RQLDAMTPDG YSLVANQNGK -GWIDPQTGH APTAADFK
WP_051963486       MTYAEQIVVT RGDQA-LVID GVSEQSKGDL KVSLGG-D-G YALDAAHDDG LVINENDSAS SGWTSSITGA AVGQADFNLT KPGAEGLREA
WP_056613648       VYFASSVAIT KGDQA-IEVT GLSQQKLGDL SITMGG-D-G RALDRANADG FVLHENDSGA -GWRSAYTGE VATQADLNLT KPGQIFGPGS
    ODB99281       MYVANELTIT KGDKV-IQVT GLSQNEVGDM QINQSDRG-G QLMDLLVTDG FVVSENACGE -GWINPETGE MATQEDFNVT KPGAEKPYE
WP_082532857       MTLASKVAIT NGDYG-VEIS GVDTNKVGDL KIDEAAGW-G AVLDWTHKDG NVLQENPAGA GFLGVDANGD IHKVDQAYMN KTDLQLNPGL
WP_090449629       EFYSSKLTIT QGDQA-IVVD HLNQNSTQPL SIINTPIL-G RLLDWATDDG TRVYEDTNTR QWVQLDAGGW THAIDGNFL
```

b

```
                73                                          116
WP_006958402      SYE-PSSGK ATLE-NDRYT INIDESSSEI EVIDKQNPED SFRIYGDPHF DI--GNDGDT DFDFKKDMSI ELDDGTKLHI HTTPT-S-NG
WP_011084615 N  WTHE-VKDGK ATINLGDKYT ITANEKDGTW TVRNNQTGHV T-VIHGDPHV DA--DGDGKD DFDFKKDMTF QLDDGTKITV NNVDY-G-NG
WP_011084464 N  WTHE-VKDGK ATINLGDKYT ITANEKDGTW TVRNNQTGHV T-KIHGDPHV DA--NGDGKD DFDFKKDMTF QLDDGTKITV NNVDY-G-NG
WP_057850284 N  WTHE-VHDGK AAIHLGDKYT ITADEKDGTW TVRNNETGHV T-KIHGDPHF DA--DGDGKD DFDFKKGMTL KLDDGTKITV DTADY-G-NG
WP_011084464 C  WSHE-VKDGK ATINLGDKYT ITADEKDGTW TVRNNQTGHV S-RIHGDPHV DA--NGDGKD DFDFKKGMTF QLDDGTKITV DTVNY-G-KG
WP_057850284 C  WSHE-VHDGK ATIHLGDKYT ITADEKDGTW TIRNKETGHV S-KIHGDPHV DA--NGDGKD DFDFKKGMTL QLDDGTKITV DTVDY-G-KG
WP_011084615 C  WSHE-VRDGK AEIKLGDKYS ILVDENDGTV LIRNSQTGKI T-SIKGDPHV DA--DGDGKV DFDFKKNMTF QLDDGTKITV DTVDI-G-KG
    OHV14582    WSHE-VKDGE ATIQLGDKYT VKASEKDATW TVTNNETGNT T-KVSGDPHV DI--DNDGKN DFDFKKDMTF KLDDGTKITV GTVPG-GENG
WP_007462645    WSVE-GNQ-- --IKLDNGYV VTVGNENRDW TITD-AAGNT T-KIWGDPHV DE--NGDG-T DWDFKQNVTF ALDDGTKISV GTAER-G-YA
WP_081833258    WTATPVTNNK ASIDLG-NYK LDFNKSDSSM TMTSKSSGDT T-KIWGDPHL TQHANGANSS TAMFNGPMTF QLPDNTKVTV GTQADKNNKS
    ACD19565    WSNTQVNDNK STIDLG-NYK LDLNKKDSSM LLTDKKSGET T-KVWGDPHI D-----SNGT SNMFNGPLSL NLSDGTKITV GTQGKGN---
WP_051963486    FTAAMPESGK AEIDLGDGNT LSINEASSEV VIRDAD-GNA T-RIWGDPHV SY--NG--KQ VGDFWGTTTF VLENGTKITI NTETSKWN-N
WP_056613648    WTVKTGTEGK ASIDLGDGYS LQLNENNSEM TITNANTGET T-RIWGDPHV DV--NG--QH VYDFWGTTTF TLDNGTKITI NTEQGQGNPN
    ODB99281    WTVCQQENGR ATIDLGDKYE LELNENKSQM IIRNKANGEE T-NIWGDPHV DW--NGDGKT DVDFWGKTTF QLEDGTKITI DTEKWKGNEN
WP_082532857     HASTQMKGG QAVFENDNYR ITAGDNNT-V NIFNKKTGET Y-NIWGDPHV NI--DG--KH AFDFYGTTTF KLEDGTKVTI ETTPWNAGNG
WP_090449629    WNVEANDGQH ARIDLG-HYT LDIDQSSSQF VLTNKATGEV T-RIWGDPHF DN--NG--QA IGTFKGTLTL SLDDGTKLTI NTTP--AGNG

                              172                                                         223                  243
WP_006958402    ETLATSLAIE EPDGSGWYIE GIDSDQKGDL EVK-EYNNIN YSGGTVNDDA ALELQVRDGN -YFLNSDNGW ETLEEDKKAS NDAIVNDIET
WP_011084615 N  QAISSKLTIT NGHNA-IVVE GLGDDKDGKN NLRVTQSNAG MTLDELTSDG AQTIHES-GQ -GWVDGAGRA VNQASIDDGE QGRGPGNYQ
WP_011084464 N  ETISSRLTIT NGHNA-MVVE GLGDDKDGKN NLRVTQSNAG MTLDELTPDG AQTIHES-GQ -GWVDGAGGV VNQASIDAGE QGRAPGNYQ
WP_057850284 N  KSISSKLTIT NGSNA-MVVE GLGDDKDGAN NLKVTQSNAG LTLDELTADG SQTIHEQ-GQ -GWVDGAGRE VDQASIDAGE EGRAPGAGS
WP_011084464 C  KTISSKLTIT NGDNA-MVVE GLGDDKDGKN NLKVTQSNAG RTLDQLTSDG AQTIHET-NQ -GWVDNSGRK VTQSVINHNE NPDAPPDFK
WP_057850284 C  KTISSKLTIT NGDDA-MVVE GLGDKKDGAN NLKVTQSNAG RTLDQLTADG AQTIYEQ-DQ -GWVDRSGWH VNQASIDANE QAA
WP_011084615 C  KTMASKLTIT NGDNA-MVVE GLGDRFDGKN NLKVTQSNAG RTLDQLTSDG AQTIYEQPGS -GWVDRSGRQ VNQEIIDSNE NPGTTSDA
    OHV14582    TTFSSSLTIT NGDKA-MQVT GLGDSHDGEN NLEVKQSDAG TTLDELENDG SATVYEN-GG -AWQTKDGKA VDQSIIDAAE AAAA
WP_007462645    NTVTDSLTIT KGNQH-IQVT GIADNNPTIS QPKLD----G LEVDAATNDG TVFYANGSTN -SWFTDKESQ NRVTSNQAMG SQQVINEDDT
WP_081833258    VSYADSVTIT HGNDA-YQVT GLSQQNSTGL SVQKSH-D-G RALDAATPDG YTLVANRNGS -GFVDPKTGK QPTEDQIRK
    ACD19565    VSYADKLTIT KGNDA-YLVN GLSEKDSNPL TVQHAG-N-G RQLDAMTPDG YSLVANQNGK -GWIDPQTGH APTAADFK
WP_051963486    MTYAEQIVVT RGDQA-LVID GVSEQSKGDL KVSLGG-D-G YALDAAHDDG LVINENDSAS SGWTSSITGA AVGQADFNLT KPGAEGLREA
WP_056613648    VYFASSVAIT KGDQA-IEVT GLSQQKLGDL SITMGG-D-G RALDRANADG FVLHENDSGA -GWRSAYTGE VATQADLNLT KPGQIFGPGS
    ODB99281    MYVANELTIT KGDKV-IQVT GLSQNEVGDM QINQSDRG-G QLMDLLVTDG FVVSENACGE -GWINPETGE MATQEDFNVT KPGAEKPYE
WP_082532857    MTLASKVAIT NGDYG-VEIS GVDTNKVGDL KIDEAAGW-G AVLDWTHKDG NVLQENPAGA GFLGVDANGD IHKVDQAYMN KTDLQLNPGL
WP_090449629    EFYSSKLTIT QGDQA-IVVD HLNQNSTQPL SIINTPIL-G RLLDWATDDG TRVYEDTNTR QWVQLDAGGW THAIDGNFL
```

Table S1. Plasmids and oligonucleotides used in this study.

Plasmid	Characteristics	Reference
pVCD019	Encoding a fusion protein consisting of MalE, the MIIA domain of the hypothetical protein VIC_001052 (amino acids 73-243 of the full-length protein) and a C-terminal Strep-tag (wt-MIIA). The MalE part can be removed by thrombin cleavage	Ibe et al 2015
pVCD019Flag	Derived from pVCD019, Strep-tag coding sequence replaced by a FLAG-tag coding sequence using oligonucleotides vcd_flagf1 and vcd_flagr1	This work
pVCD020	Derived from pVCD019 encoding a MIIA domain with a D116A replacement (D116A-MIIA) constructed by using oligonucleotides D116Afor and D116Arev	This work
pVCD042	Derived from pVCD019 encoding a truncated MIIA domain (MIIA73-223) constructed by using oligonucleotides vcd_c20f and vcd_c20r	This work
pVCD043	Derived from pVCD019 encoding a truncated MIIA domain (MIIA73-218) constructed by using oligonucleotides vcd_c25f and vcd_c25r	This work
pVCD046	Derived from pVCD019Flag, encoding a MIIA domain with a W172F replacement (W172F-MIIA) constructed by using oligonucleotides Trp 1_for and Trp1_rev	This work
pVCD047	Derived from pVCD019Flag, encoding a MIIA domain with a W223F replacement (W223F-MIIA) constructed by using oligonucleotides Trp 2_for and Trp2_rev	This work

Oligonucleotide	Sequence	Usage
vcd_flagf1	GATATTGAAACCCTCGAGGACTACAAGGACGACGATGACAAGTGACTAGTGCGGCCGCATC	Generation of pVCD019Flag
vcd_flagr1	GATGCGGCCGCACTAGTCACTTGTCATCGTCGTCCTTGTAGTCCTCGAGGGTTTCAATATC	Generation of pVCD019Flag
D116Afor	GAAGATAGCTTTCGCATTTATGGAGCTCCGCATTTTGATATTGGCAATG	Generation of pVCD020
D116Arev	CATTGCCAATATCAAAATGCGGAGCTCCATAAATGCGAAAGCTATCTTC	Generation of pVCD020
vcd_c20f	CTGAATAGCGATAATGGCTGGCTCGAGTGGAGCCACCCGCAGTTCG	Generation of pVCD042
vcd_c20r	CGAACTGCGGGTGGCTCCACTCGAGCCAGCCATTATCGCTATTCAG	Generation of pVCD042
vcd_c25f	CGTGATGGTAATTATTTCTGAATCTAGAGTGGAGCCACCCGCAGTTCG	Generation of pVCD043
vcd_c25r	CGAACTGCGGGTGGCTCCACTCTAGATTCAGAAAATAATTACCATCACG	Generation of pVCD043
Trp 1_for	GCCTGGCAATTGAAGAACCGGATGGCTCGGGTTTTTATATTGAAGGTATTGATAGC	Generation of pVCD046
Trp 1_rev	GCTATCAATACCTTCAATATAAAAACCCGAGCCATCCGGTTCTTCAATTGCCAGGC	Generation of pVCD046
Trp 2_for	CTGAATAGCGATAATGGCTTTGAAACCCTCGAGGAAGATAAAAAAGCAAGC	Generation of pVCD047
Trp 2_rev	GCTTGCTTTTTTATCTTCCTCGAGGGTTTCAAAGCCATTATCGCTATTCAG	Generation of pVCD047

Table S2. Sequences in the wt-MIIA domain with highest similarity to EF-hand Ca^{2+}-binding loops. Using the Expasy online tool Scan Prosite(http://prosite.expasy.org/scanprosite/) the original Prosite pattern PS00018 for Ca^{2+}-binding loops in EF-hand motifs and the patterns 1 and 2 derived from it by the indicated reduction of sequence constraints reveal Ca^{2+}-binding loop-like sequences. #matches: number of amino acids in the retrieved sequences that match the constraints of the original strict EF-hand pattern. Only the sequence that was identified in addition to the more restrictive pattern 1 is shown for pattern 2. Capital letters indicate amino acids that comply with constraints in the original Prosite pattern PS00018 or correspond to a conservative replacement.

Pattern identifier	sequence pattern (constraints different from PS00018 are highlighted)	number of matches with PS00018	sequences found in wt-MIIA domain
PS00018	D-{W}-[DNS]-{ILVFYW}-[DENSTG]-[DNQGHRK]-{GP}-[LIVMC]-[DENQSTAGC]-x(2)-[DE]	10	none
Pattern-1	D-{W}-[DNS]-{ILVFYW}***-X-***[DNQGHRK]-{GP}-[LIVMC]-[DENQSTAGC]-x(2)-[DE]	9	^{178}DSDQkGDLEvkE^{189}
Pattern-2	[D**E**]-{W}-[DNS]-{ILVFYW}-***X***-[DNQGHRK]-{GP}**-X(2)-**x(2)-[DE]	7	^{75}EPSSGKAtlenD^{86}

Reference

1. Prilusky, J., C.E. Felder, T. Zeev-Ben-Mordehai, E. Rydberg, O. Man, J.S. Beckmann, I. Silman & J.L. Sussman. FoldIndex©: a simple tool to predict whether a given protein sequence is intrinsically unfolded. *Bioinformatics* (2005).
2. Access to PONDR® is provided by Molecular Kinetics (6201 La Pas Trail - Ste 160, Indianapolis, IN 46268; (www.molecularkinetics.com; main@molecularkinetics.com) under license from the WSU Research Foundation. PONDR® is copyright ©1999 by the WSU Research Foundation, all rights reserved.
3. Whitmore, L. & Wallace, B. A. Protein secondary structure analyses from circular dichroism spectroscopy: Methods and reference databases. *Biopolymers* **89**, 392-400 (2008).
4. Whitmore, L. & Wallace, B. A. DICHROWEB, an online server for protein secondary structure. *Nucleic Acids Res* **32**, W668-73 (2004).
5. Schirrmeister, J., S. Zocher, L. Flor, M. Göttfert & S. Zehner. The domain of unknown function DUF1521 exhibits metal ion-inducible autocleavage activity - a novel example from a putative effector protein of Vibrio coralliilyticus ATCC BAA-450. *FEMS Microbiol Lett* **343**, 177-182 (2013).
6. Corpet, F. Multiple sequence alignment with hierarchical clustering. Nucleic Acids Res **16**, 10881-10890 (1988).
